# Safety, Tolerability, and Immunogenicity of an mRNA-Based Respiratory Syncytial Virus Vaccine in Healthy Young Adults in a Phase 1 Clinical Trial

**DOI:** 10.1093/infdis/jiae035

**Published:** 2024-01-31

**Authors:** Christine A Shaw, Runa Mithani, Archana Kapoor, Rakesh Dhar, Lauren Wilson, Laila El Asmar, Sabine Schnyder-Ghamloush, Kristi Schaefers, Allison August, Sonia K Stoszek, Grace L Chen

**Affiliations:** Moderna, Inc, Cambridge, Massachusetts, USA; Moderna, Inc, Cambridge, Massachusetts, USA; Moderna, Inc, Cambridge, Massachusetts, USA; Moderna, Inc, Cambridge, Massachusetts, USA; Moderna, Inc, Cambridge, Massachusetts, USA; Moderna, Inc, Cambridge, Massachusetts, USA; Moderna, Inc, Cambridge, Massachusetts, USA; Moderna, Inc, Cambridge, Massachusetts, USA; Moderna, Inc, Cambridge, Massachusetts, USA; Moderna, Inc, Cambridge, Massachusetts, USA; Moderna, Inc, Cambridge, Massachusetts, USA

**Keywords:** mRNA-1345, mRNA vaccine, respiratory syncytial virus, safety and immunogenicity, younger adult

## Abstract

**Background:**

Respiratory syncytial virus (RSV) presents a global health concern. A lipid nanoparticle–encapsulated mRNA-based RSV vaccine (mRNA-1345) that encodes the membrane-anchored RSV prefusion–stabilized F glycoprotein is under clinical investigation.

**Methods:**

This phase 1 dose escalation study was based on a randomized, observer-blind, placebo-controlled design, and it assessed the safety and immunogenicity of mRNA-1345 in healthy adults aged 18 to 49 years. Participants were randomized to receive 1 dose of mRNA-1345 (50, 100, or 200 µg) or placebo or 3 doses of mRNA-1345 (100 µg) or placebo 56 days apart.

**Results:**

mRNA-1345 was well tolerated at all dose levels. The most common solicited adverse reactions were pain, headache, fatigue, myalgia, or chills, which were all generally mild to moderate. At 1 month postinjection, a single injection of mRNA-1345 boosted RSV neutralizing antibody titers (geometric mean fold rise: RSV-A, 20.0–23.5; RSV-B, 11.7–16.0) and RSV prefusion binding antibody concentrations (geometric mean fold rise, 16.1–21.8), with no apparent dose response. Antibody levels remained above baseline through 6 months. Sequential doses of 100 µg were well tolerated but did not further boost antibody levels.

**Conclusions:**

A single mRNA-1345 injection demonstrated an acceptable safety profile in younger adults and induced a durable neutralizing antibody response, supporting its continued development.

**Clinical Trials Registration:**

ClinicalTrials.gov NCT04528719.

## Teaser

Respiratory syncytial virus presents a global health concern. We assessed the safety and immunogenicity of an mRNA-based vaccine, mRNA-1345, in healthy adults aged 18 to 49 years. mRNA-1345 vaccine was well tolerated and immunogenic in younger adults.

Globally, respiratory syncytial virus (RSV) is a common cause of lower respiratory tract disease in infants, children, and adults with chronic comorbidities, as well as older adults and individuals who are immunocompromised [1–4]. Most become infected early in life, and reinfections throughout life are common [3, 5]. Worldwide, an estimated 33 million cases of RSV-associated acute lower respiratory tract infections occur in children aged <5 years, leading to 3.6 million hospitalizations and 26 300 hospital deaths annually [6]. In 2019, there were approximately 5.2 million RSV cases, leading to around 470 000 hospitalizations and 33 000 in-hospital deaths in high-income countries among adults aged ≥60 years [7].

There is no specific treatment for RSV disease in adults [1, 8]. Two RSV monoclonal antibodies, palivizumab and nirsevimab, are approved as RSV prophylaxis in infants [9–14]. After decades of RSV vaccine development, safe and effective RSV vaccines are on the horizon for older adults but not yet for active vaccination of children [15–17]. Notably, in clinical trials in the 1960s, a formalin-inactivated RSV vaccine resulted in enhanced respiratory disease in infants and children previously RSV naive following an RSV infection [18, 19].

Improved understanding of the RSV envelope F protein structure [20] has recently led to vaccine development predominantly focused on the F protein to optimize antibody responses [13, 14, 21–23]. The F protein is the main antigenic target of protective neutralizing antibodies [21] and is conserved across strains and antigenic subtypes (RSV-A and RSV-B) [22]. The F protein exists in 2 primary conformational states: prefusion (preF) and postfusion (postF). The metastable preF state drives virus and host cell-membrane fusion through a conformational change to the stable postF state. The preF conformation displays all epitopes known to elicit neutralizing antibodies and induces higher neutralizing antibody responses than the postF conformation in animal models and humans [20, 23–25].

The mRNA vaccine platform offers multiple advantages over conventional approaches, including rapid and scalable manufacturing and ease of adaption to new antigen designs [26, 27]. The safety and efficacy of the mRNA-based COVID-19 vaccine mRNA-1273 (Spikevax; Moderna) [28–31] has established the utility of such a platform for the development of other mRNA-based vaccines against respiratory pathogens, including influenza [32], human metapneumovirus, and parainfluenza virus type 3 [33], and newer iterations of the COVID-19 vaccine [34]. Notably, phase 1 data from these vaccines demonstrated an acceptable safety profile and induction of humoral and cell-mediated immune responses, making this platform ideally suited for RSV vaccine development. We previously described mRNA-1777: an mRNA-based RSV vaccine candidate encoding the membrane-associated RSV F glycoprotein stabilized in the preF conformation [35]. Here, we describe another mRNA-based RSV vaccine candidate, mRNA-1345, also encoding the membrane-associated RSV F protein stabilized in the preF conformation but optimized through mRNA, protein, and formulation design.

Herein, we report the findings from a phase 1, first-in-human, dose escalation study that assessed the safety, tolerability, and immunogenicity of different dose levels and regimens of mRNA-1345 in healthy adults.

## METHODS

### Study Design and Participants

**Figure 1.**
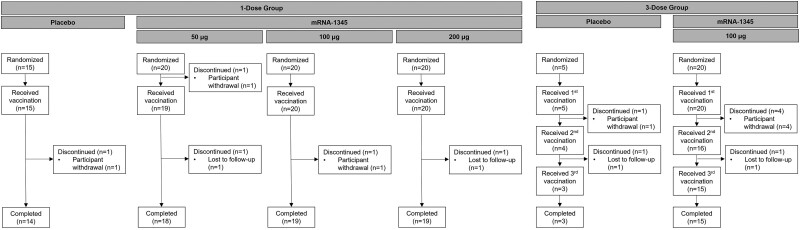
Disposition of participants aged 18 to 49 years in the 1- and 3-dose cohorts.

This randomized, observer-blind, placebo-controlled study of healthy adults aged 18 to 49 years was conducted in the United States (ClinicalTrials.gov, NCT04528719; see supplementary methods for details). Within each of the dose-level/regimen cohorts of 25 participants, individuals were randomly assigned 4:1 via an interactive response technology system to receive 1 dose of mRNA-1345 (50, 100, or 200 μg) or placebo or 3 doses of mRNA-1345 (100 μg) or placebo 56 days apart (Figure 1). A total of 60 participants received 1 dose of mRNA-1345, 20 received 1 dose of placebo, 20 received 3 doses of mRNA-1345, and 5 received 3 doses of placebo. Participants were followed for 6 months after the last injection. The protocol was approved by a central institutional review board, and the study was conducted according to the principles of the International Council for Harmonisation’s good clinical practice. All aspects of the study were performed in accordance with the ethical principles originating in the Declaration of Helsinki and the protocol, as well as all national, state, and local laws or regulations. All participants provided written informed consent, which was obtained before study entry and performance of procedures. Full eligibility criteria are included in the supplementary methods. This study is part of a larger ongoing phase 1 study that includes women of childbearing potential aged 18 to 40 years, healthy adults aged 65 to 79 years, adults of Japanese descent aged ≥60 years, and children aged 12 to 59 months who were RSV seropositive.

### Vaccine

mRNA-1345 contains a single nucleoside-modified mRNA sequence encoding the membrane-anchored RSV F glycoprotein (RSV-A2 strain protein sequence) stabilized in the preF conformation through structural engineering and formulated in lipid nanoparticles (LNPs). The LNP formulation consists of an ionizable lipid promoting assembly of LNPs into delivery vehicles, a phospholipid that forms lipid bilayer structures in LNPs, a polyethylene glycol lipid, and a sterol that improves the stability of the formulations [27, 36]. mRNA-1345 was provided as a lyophilized drug product and reconstituted with 0.9% sodium chloride to form a uniform 0.8-mg/mL mRNA-LNP dispersion. The vaccine was prepared by diluting to yield a final dose volume of 0.5 mL for each dose level. Following dose preparation, 0.5 mL of mRNA-1345 or placebo (0.9% sodium chloride) was administered by intramuscular injection into the deltoid muscle on day 1 (1-dose regimen) or days 1, 57, and 113 (3-dose regimen).

### Study Objectives

The primary objective was to evaluate the safety and tolerability of mRNA-1345 in younger adults: 1 injection at 3 dose levels (50, 100, or 200 µg) and 3 injections at the 100-µg dose level given 56 days apart. The secondary objective was to evaluate immunogenicity following mRNA-1345 vaccination.

### Safety Assessments

Safety assessments included solicited local and systemic adverse reactions (ARs), unsolicited adverse events (AEs), medically attended AEs, serious AEs, AEs of special interest (Supplementary Table 1), and AEs leading to study withdrawal. Solicited ARs were considered to be related to study injection and were collected within 7 days of injection, and unsolicited AEs were collected within 28 days of injection. Medically attended AEs and serious AEs were collected throughout the study.

Solicited local ARs of injection site pain, erythema, and swelling/induration were assessed, as well as solicited systemic ARs of fever, headache, fatigue, myalgia, arthralgia, nausea-vomiting, lymphadenopathy, and chills. Solicited ARs that occurred during the 7 days following injection were recorded by the participants via an eDiary.

Correlation of immune or other laboratory parameters with the frequency and/or severity of AEs was not evaluated.

### Immunogenicity Assessments

Blood samples were collected prior to study product administration on day 1, monthly from months 1 through 6, and at month 10 for the 3-dose cohorts only. Immunogenicity was assessed by measuring serum neutralizing antibody titers against RSV–A and RSV–B and binding antibody concentrations against the RSV F protein (preF and postF conformations).

RSV-A and RSV-B neutralizing antibodies were measured in qualified microneutralization assays (supplementary methods), and results are presented as geometric mean titer (international units per milliliter, per the World Health Organization international standard for antiserum to RSV) and geometric mean fold rise (GMFR; defined as the geometric mean ratio of postbaseline/baseline titers.

The preF and postF binding (immunoglobulin G) antibodies were measured in a qualified multiplex Luminex assay (supplementary methods), and results are presented as geometric mean concentration (arbitrary units per milliliter) and GMFR.

### Data Analysis

There was no formal hypothesis testing or inferential statistical analyses across vaccination arms. A sample size of 100 adults was planned for study enrollment and randomization, which was considered sufficient to provide a descriptive summary of the tolerability and immunogenicity of different dose levels of mRNA-1345. Demographic variables (eg, age, height, weight, and body mass index) and baseline characteristics were descriptively summarized by vaccination arm and overall (mean and SD for continuous variables, number and percentage for categorical variables). Safety analyses were descriptive and presented by vaccination arms. Immunogenicity data were summarized at each time point according to the per-protocol set, including geometric mean titer (neutralizing), geometric mean concentration (binding), and GMFR. The corresponding 95% CI was calculated per the t-distribution of the log-transformed value and then back transformed to the original scale. Analyses were performed with SAS version 9.4 (SAS Institute). Sample size calculations and analysis populations are described in the supplementary methods.

## RESULTS

### Participants

One hundred participants were enrolled and 79 (80%) received mRNA-1345. An overall 88 participants (88%) completed the study, and of these, 71 (81%) received mRNA-1345 (Figure 1). One participant in the 50-μg group withdrew consent prior to receiving the vaccination. In the 1-dose cohorts, 1 (5%), 1 (5%), 1 (5%), and 1 (6.7%) participants in the 50-, 100-, 200-μg, and placebo groups, respectively, prematurely discontinued the study. In the 3-dose cohorts, 5 participants (25%) in the 100-μg group and 2 (40%) in the placebo group prematurely discontinued the study. Five participants (25%) in the 100-μg 3-dose group and 2 (40%) in the placebo 3-dose group also prematurely discontinued vaccination. For study and vaccination discontinuation, the reasons were attributed to loss to follow-up or withdrawal by the participant and not due to an AE or death.

Most participants were White (78%) and not Hispanic or Latino (98%); the mean age was 33.5 years (Table 1). Sex was balanced in the 50- and 100-μg 1-dose groups; however, the 200-μg group had 30% female participants and the placebo 1-dose group had 80% female participants. In the mRNA-1345 100-μg and placebo 3-dose groups, 80% and 60% female participants were enrolled, respectively. Thirty-four participants (57.6%) in the mRNA-1345 1-dose group, 2 (13.3%) in the placebo 1-dose group, 9 (45%) in the 100-μg 3-dose group, and 0 in the placebo 3-dose group used concomitant medication though 7 days postvaccination.

**Table 1.** Baseline Demographics

	1-Dose Group^a^				3-Dose Group^a^		
	Placebo (n = 15)	50 µg (n = 20)	100 µg (n = 20)	200 µg (n = 20)	Placebo (n = 5)	100 µg (n = 20)	Overall (N = 100)
Age, y	37.5	36.1	33.4	31.8	24.8	32.0	33.5
Sex							
Female	12 (80.0)	10 (50.0)	11 (55.0)	6 (30.0)	3 (60.0)	16 (80.0)	58 (58.0)
Male	3 (20.0)	10 (50.0)	9 (45.0)	14 (70.0)	2 (40.0)	4 (20.0)	42 (42.0)
Race							
White	11 (73.3)	14 (70.0)	16 (80.0)	18 (90.0)	3 (60.0)	16 (80.0)	78 (78.0)
Black or African American	4 (26.7)	6 (30.0)	4 (20.0)	0	2 (40.0)	4 (20.0)	20 (20.0)
Multiple	0	0	0	2 (10.0)	0	0	2 (2.0)
Ethnicity							
Hispanic or Latino	1 (6.7)	0	1 (5.0)	0	0	0	2 (2.0)
Not Hispanic or Latino	14 (93.3)	20 (100.0)	19 (95.0)	20 (100.0)	5 (100.0)	20 (100.0)	98 (98.0)
Body mass index, kg/m^2^	28.61	27.60	27.87	24.81	28.26	27.28	27.21
Any concomitant medication within 7 d postvaccination	2 (13.3)	9 (47.4)	6 (30.0)	19 (95.0)	0	9 (45.0)	45 (45.0)

Data are presented as mean or No. (%).

^a^50-, 100-, and 200-μg doses of the mRNA-1345 vaccine.

### Safety

**Figure 2.**
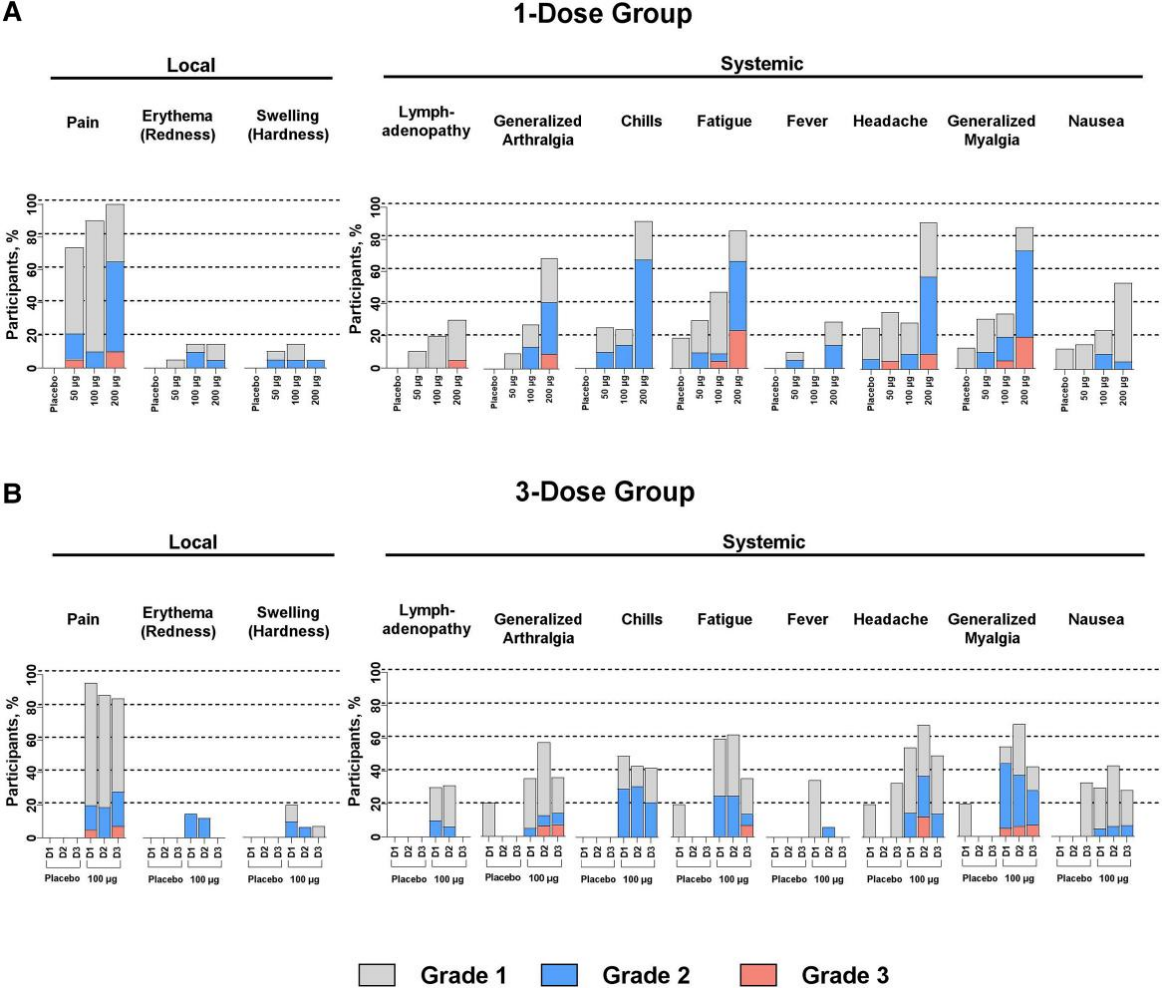
Solicited (*A*) 1-dose and (*B*) 3-dose local and systemic adverse reactions within 7 days of receiving each dose. D1, dose 1; D2, dose 2; D3, dose 3.

The incidence and severity of solicited local and systemic ARs were dose dependent in the mRNA-1345 groups (Figure 2).

#### Solicited Local ARs

After the first injection, solicited local ARs were most frequent in participants receiving 200-µg mRNA-1345 (100%) or 100-µg mRNA-1345 (90.0%, 1-dose group; 95.0%, 3-dose group) as compared with participants receiving 50-µg mRNA-1345 (73.7%). After the second and third injections of 100-µg mRNA-1345, 87.5% and 85.7% of participants experienced local ARs, respectively. No local ARs were reported in the placebo groups.

The most common local solicited AR after the first injection was injection site pain, reported by 100% of participants in the 200-µg 1-dose group, 90.0% in the 100-µg 1-dose group, 95.0% in the 100-µg 3-dose group, and 73.7% in the 50-µg 1-dose group. These ARs were mostly grade 1 or 2 in severity, with grade 3 pain in 10.0% and 5.3% of the 200- and 50-µg groups, respectively. The median duration of pain in the mRNA-1345 group was 2.0 to 3.0 days.

Pain at the injection site was the most common local solicited AR after the second and third 100-µg mRNA-1345 injections, reported by 87.5% and 85.7% of participants, respectively. These ARs were mostly grade 1 or 2 in severity, with no grade 3 pain after the second injection and 1 case (7.1%) of grade 3 pain after the third injection (Supplementary Table 2). The median duration of pain in the mRNA-1345 group was 3.0 days after the second and third injections.

#### Solicited Systemic ARs

After the first injection, solicited systemic ARs were most frequent in participants receiving 200-µg mRNA-1345 (100%) or 100-µg mRNA-1345 (70.0%, 1-dose group; 90.0%, 3-dose group) as compared with participants receiving 50-µg mRNA-1345 (57.9%) or placebo (40.0%, 1-dose group; 40.0%, 3-dose group; Figure 2). After the second and third injections in the 3-dose group, 81.3% and 64.3% of participants in the 100-µg mRNA-1345 group and 0% and 33.3% in the placebo group reported systemic ARs, respectively.

Overall, 76.3% of participants had at least 1 systemic solicited AR in the 1-dose vaccine groups. These were primarily headache, fatigue, myalgia, or chills, with the highest rates in the 200- and 100-μg groups. Grade 3 solicited systemic ARs were generally dose dependent, occurring in 5.3%, 5.0%, and 30.0% of participants in the 50-, 100- (1-dose), and 200-μg groups, respectively. The most common solicited systemic AR after the first injection was fatigue, reported by 90.0% of participants in the 200-µg 1-dose group, 50.0% in the 100-µg 1-dose group, 60.0% in the 100-µg 3-dose group, and 31.6% in the 50-µg 1-dose group. The median duration of fatigue was 1.5 to 2.0 days in the mRNA-1345 group and 1.0 days in placebo group. No increase in overall ARs was noted after dose 2 or 3 of the 3-dose mRNA-1345 regimen as compared with dose 1.

#### Unsolicited AEs

In the 1-dose placebo group, 33.3% of participants reported unsolicited AEs (Supplementary Table 3). Unsolicited treatment-emergent AEs (TEAEs) occurred in 10.5%, 10.0%, 30.0%, 75.0%, and 33.3% of participants in the 1-dose 50-µg, 1-dose 100-µg, 3-dose 100-µg, 1-dose 200-µg, and 1-dose placebo groups, respectively. After the second and third injections in the 3-dose 100-µg group, 6.3% and 20.0% of participants reported unsolicited AEs. The most frequent unsolicited TEAEs (≥10%) included dyspnea, headache, injection site pain, aphthous ulcer, nasopharyngitis, and nausea.

Unsolicited treatment-related TEAEs occurred in 10.5%, 5.0%, 15.0%, and 0% of participants in the 50-, 100-, 200-µg, and placebo groups of the 1-dose vaccine groups, respectively (Table 2). Ten percent and 6.3% of participants in the 3-dose 100-µg group reported treatment-related TEAEs after the first and second injections. No unsolicited treatment-related TEAEs were noted after any injection in the 3-dose placebo group or after the third 100-µg mRNA-1345 injection. There were no unsolicited treatment-related serious AEs, AEs of special interest, medically attended AEs, fatal TEAEs, TEAEs leading to study discontinuation, or grade ≥3 TEAEs; review of unsolicited AEs of special interest did not identify reports of anaphylactic reaction, myocarditis/pericarditis, Guillain-Barré syndrome, Bell palsy/facial paralysis, acute demyelinating encephalomyelitis, seizures, or thrombocytopenia associated with mRNA-1345 administration.

**Table 2.** Unsolicited Treatment-Related Adverse Events: Exposed Set

	1-Dose Group^a^				3-Dose Group^a^					
					Placebo			100 μg		
	Placebo (n = 15)	50 μg (n = 19)	100 μg (n = 20)	200 μg (n = 20)	Dose 1 (n = 5)	Dose 2 (n = 4)	Dose 3 (n = 3)	Dose 1 (n = 20)	Dose 2 (n = 16)	Dose 3 (n = 15)
All unsolicited TEAEs	0	2 (10.5)	1 (5.0)	3 (15.0)	0	0	0	2 (10.0)	1 (6.3)	0
SAEs	0	0	0	0	0	0	0	0	0	0
MAAEs	0	0	0	0	0	0	0	0	0	0
AESIs	0	0	0	0	0	0	0	0	0	0
Fatal TEAEs	0	0	0	0	0	0	0	0	0	0
TEAEs leading to study discontinuation	0	0	0	0	0	0	0	0	0	0
TEAEs grade ≥3	0	0	0	0	0	0	0	0	0	0

Exposed set: all participants in the randomized set who received any study vaccination. Data are presented as No. (%).

Abbreviations: AESI, adverse event of special interest; MAAE, medically attended adverse event; SAE, serious adverse event; TEAE, treatment-emergent adverse event.

^a^50-, 100-, and 200-μg doses of the mRNA-1345 vaccine.

### Immunogenicity

#### Neutralizing and Binding Antibody Response Following Vaccination

**Figure 3.**
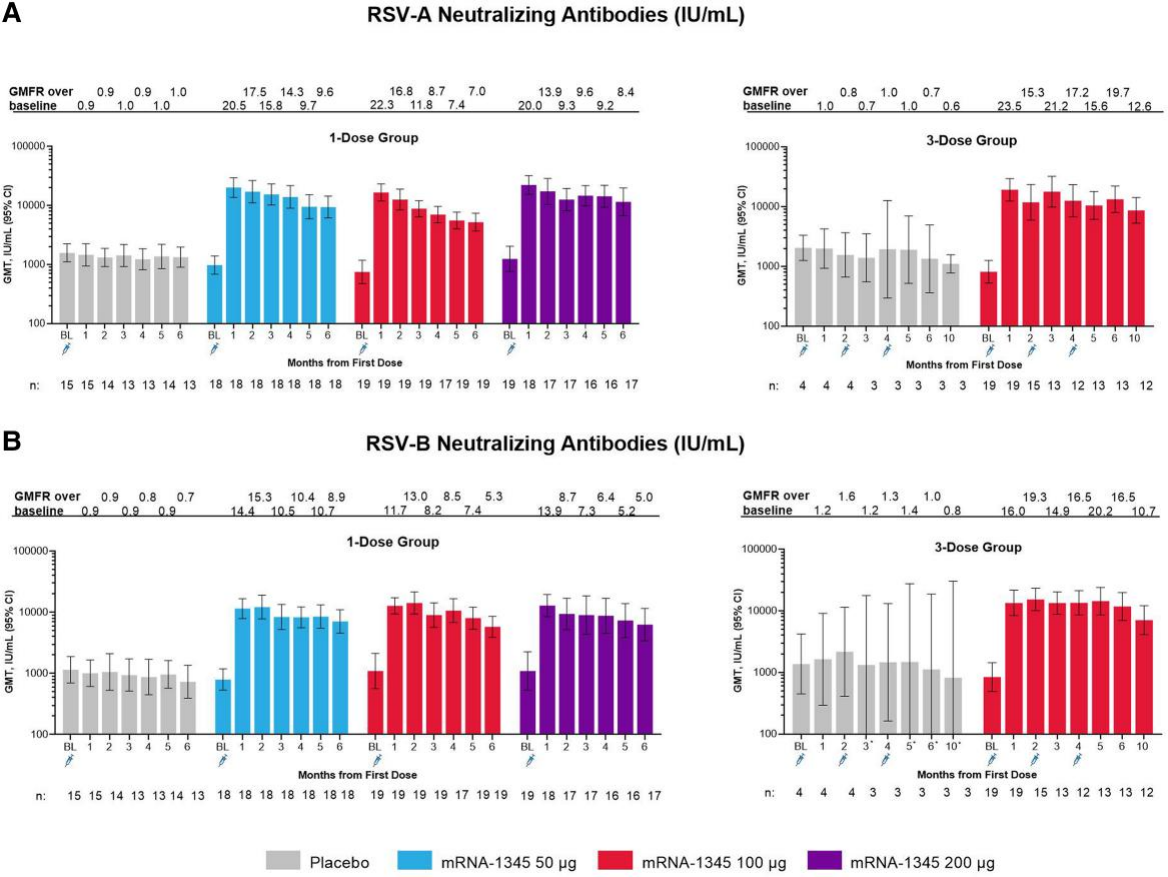
Serum neutralizing antibody GMTs and GMFR against time for the 1- and 3-dose groups against (*A*) RSV-A and (*B*) RSV-B. *In the mRNA-1345 100-µg 3-dose placebo group, lower-bound 95% CIs of 98.6, 79.2, 66.8, and 22.2 were noted at months 3, 5, 6, and 10, respectively. BL, baseline; GMFR, geometric mean fold rise; GMT, geometric mean titer; RSV, respiratory syncytial virus.

**Figure 4.**
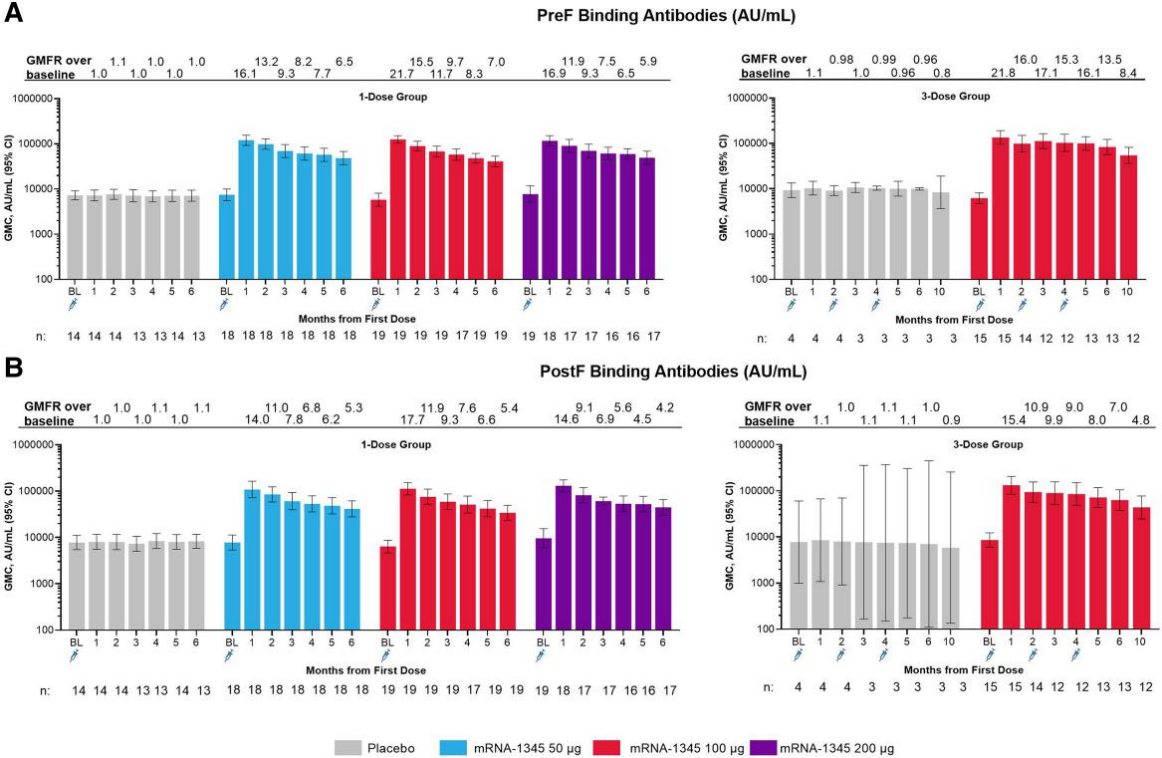
Serum binding antibody GMC and GMFR against time for the 1- and 3-dose groups for (*A*) preF and (*B*) postF binding antibodies. GMC, geometric mean concentration; GMFR, geometric fold rise; postF, postfusion; preF, prefusion; RSV, respiratory syncytial virus.

Neutralizing antibodies (international units per milliliter) against RSV-A and RSV-B and binding antibodies (arbitrary units per milliliter) against preF and postF were present at baseline, consistent with prior exposure to RSV (Figures 3 and 4). One mRNA-1345 injection of 50, 100, or 200 μg increased RSV-A and RSV-B neutralizing antibody titers (Figure 3, Supplementary Table 4), as well as preF and postF binding antibody concentrations (Figure 4, Supplementary Table 5). No apparent dose response was observed. For the mRNA-1345 groups, RSV-A neutralizing antibody geometric mean titers were 750 to 1253 at baseline and 16 688 to 22 312 at month 1; RSV-B neutralizing antibody geometric mean titers were 795 to 1097 at baseline and 11 488 to 13 497 at month 1. Month 1 GMFRs ranged from 20.0 to 23.5 for RSV-A and 11.7 to 16.0 for RSV-B. The month 1 GMFR for placebo was 0.9 to 1.0 and 0.9 to 1.2 for RSV-A and RSV-B, respectively. An exploratory analysis showed an inverse relationship between baseline RSV-A or RSV-B neutralizing antibody titers and the GMFR 1 month after mRNA-1345 vaccination: individuals with the lowest baseline titers tended to have the highest GMFRs (Supplementary Figure 1). A positive relationship between RSV-A and RSV-B titers at month 1 was also observed (Supplementary Figure 2). RSV preF and postF binding antibody responses followed similar trends as RSV neutralizing antibody responses. Month 1 binding antibody GMFRs ranged from 16.1 to 21.8 for preF and 14.0 to 17.7 for postF. The month 1 GMFR for placebo was 1.0 to 1.1 for preF and postF. In all mRNA-1345 groups, the geometric mean fold change ratio of preF to postF binding antibody was >1 at month 1, suggesting that mRNA–1345 preferentially boosts antibodies to the preF conformation (Supplementary Figure 3). Additionally, the geometric mean fold change ratio of RSV neutralizing antibody to postF binding antibody was 1.4 for RSV-A and 0.9 for RSV-B, demonstrating a balanced induction of neutralizing and binding antibodies (Supplementary Figure 4).

RSV antibody levels declined from month 1 to month 6 but remained substantially above baseline. The neutralizing antibody GMFR at month 6 in the mRNA-1345 1-dose groups ranged from 7.0 to 9.6 for RSV-A and 5.0 to 8.9 for RSV-B. The binding antibody GMFR at month 6 ranged from 5.9 to 7.0 for preF and 4.2 to 5.4 for postF.

Second and third 100-μg injections at months 2 and 4 did not boost neutralizing antibody titers relative to 1 month after the first injection but maintained peak titers through 5 months after the first injection, relative to a single 100-μg injection. Antibody titers in the 100-μg 3-dose group declined slightly from month 5 to month 10 but remained substantially above baseline. The GMFR at month 10 was 12.6 for RSV-A neutralizing antibodies, 10.7 for RSV-B neutralizing antibodies, 8.4 for preF binding antibodies, and 4.8 for postF binding antibodies.

## DISCUSSION

This first-in-human trial of mRNA-1345 in healthy young adults demonstrated that mRNA-1345 was well tolerated, had an acceptable safety profile, and elicited a robust humoral immune response that was maintained through 6 months of follow-up.

mRNA-1345 was generally well tolerated at all doses, with mostly mild solicited ARs observed. A dose-response relationship was generally observed for solicited local and systemic ARs; there was no apparent relationship observed in the rate or severity of solicited ARs with the second or third injection when compared with the first injection in the 3-dose group. There were no reported serious AEs, fatal AEs, AEs of special interest, or AEs leading to vaccination or study discontinuation.

A single dose of mRNA-1345 boosted RSV-A and RSV-B neutralizing antibody titers and binding antibody concentrations at all dose levels evaluated. No dose-response relationship was observed, which suggests the potential for a low-dose vaccine. The binding antibody response was preF biased, which suggests that mRNA-1345 can induce a robust immune response to RSV preF, the main antigenic target of RSV humoral immunity [35]. Additionally, balanced induction of neutralizing and binding antibodies was observed; given that nonneutralizing antibodies have been associated with formalin-inactivated RSV–enhanced respiratory disease, the balanced response induced by mRNA-1345 may reduce the risk of enhanced respiratory disease in children who are RSV naive. Second and third 100-µg injections at months 2 and 4 did not increase antibody levels relative to the first injection; this suggests that a single-dose vaccine could produce durable immune responses in individuals who are RSV seropositive. Furthermore, in an ongoing phase 2/3 pivotal trial in adults aged ≥60 years, a single dose of mRNA-1345 met primary endpoint success criteria with 83.7% efficacy against RSV–lower respiratory tract disease with ≥2 symptoms [37]. Immunogenicity of a booster dose given 1 year (ClinicalTrials.gov, NCT04528719 and NCT05330975) or 2 years (ClinicalTrials.gov, NCT05127434) after the first vaccination is under evaluation [38–40].

The limited treatment options for RSV reinforce the need for a safe and effective vaccine for the prevention of RSV disease. In a phase 1/2 study in young adults aged 18–40 years, a recombinant RSV preF vaccine (RSVPreF3; 1 dose at 30, 60, or 120 µg) had an acceptable safety profile and boosted neutralizing antibody titers, with RSV-A titers increasing 7.5- to 13.7-fold 1 month after vaccination [41]. Additionally, a phase 1/2 study of a recombinant RSV bivalent preF vaccine (1 dose at 60, 120, or 240 µg) in adults aged 18 to 49 years demonstrated an acceptable safety profile and GMFRs 1 month postvaccination, ranging from 10.6 to 16.9 and 10.3 to 19.8 for RSV-A and RSV-B, respectively [42]. Both vaccines were also efficacious in phase 3 clinical studies in adults aged ≥60 years [16, 17]. In a previous phase 1 study by our group, a first-generation mRNA RSV vaccine (mRNA-1777; 1 dose at 25, 100, or 200 µg) in younger adults had an acceptable safety profile and peak RSV-A GMFRs 29 to 90 days postvaccination, ranging 2.5- to 3.9-fold [35]. In this trial, mRNA-1345 induced robust humoral responses (RSV-A GMFR, 20.0–23.5; RSV-B GMFR, 11.7–16.0) that were comparable or greater to the response reported for other studies. However, that direct comparison of the data is limited by differences in the population groups investigated, small sample sizes, and differences in the antibody assays used.

The strengths of this study include the randomized, observer-blind, placebo-controlled design, which controls for variability and bias. Study limitations include limited sample sizes across each dose group, a higher percentage of women in certain cohorts, and an overall higher percentage of White participants due to the population distribution at the study sites, potentially limiting generalizability. Finally, despite the absence of a dose response in the younger adult population, results may differ in other populations.

This phase 1 trial showed that mRNA-1345 is well tolerated and immunogenic in younger adults. Additionally, the data show that high antibody titers can be achieved with a low dose of mRNA-1345. Together, these data support the continued clinical development of the mRNA-1345 vaccine for the prevention of RSV disease. Notably, a phase 2/3 trial of mRNA-1345 in adults aged ≥60 years (ClinicalTrials.gov NCT05127434) is ongoing to address the urgent need for an RSV vaccine in this age group and has demonstrated efficacy against RSV–lower respiratory tract disease [37].

## Supplementary Data

Supplementary materials are available at *The Journal of Infectious Diseases* online (http://jid.oxfordjournals.org/). Supplementary materials consist of data provided by the author that are published to benefit the reader. The posted materials are not copyedited. The contents of all supplementary data are the sole responsibility of the authors. Questions or messages regarding errors should be addressed to the author.

## Supplementary Material

jiae035_Supplementary_Data

## Notes

***Acknowledgments.*** We thank the participants in the trial and the members of the phase 1 trial team for their dedication and their contributions to the trial, as well as the members of the data safety monitoring board for their hard work, support, and guidance of the trial. Medical writing and editorial assistance were provided by Jared Mackenzie, PhD, of MEDiSTRAVA, in accordance with GPP3 guidelines (Good Publication Practice), as funded by Moderna, Inc, and under the direction of the authors.

***Author contributions.*** A. K. and G. L. C. were involved in data collection. C. A. S., A. K., R. D., L. W., S. K. S., and G. L. C. analyzed or interpreted the data. All authors contributed toward the concept and design of the study; provided writing, review, or intellectual contributions; and approved the final draft.

***Data availability.*** Upon request and subject to review, Moderna, Inc, will provide the data that support the findings of this study. Moderna, Inc, may also provide access to the related individual anonymized participant data, subject to certain criteria, conditions, and exceptions.

***Financial support*.** This work was supported by Moderna, Inc. Funding to pay the Open Access publication charges for this article was provided by Moderna, Inc.
