# Supplementary material for: Safety, Tolerability, and Immunogenicity of an mRNA-Based Respiratory Syncytial Virus Vaccine in Healthy Young Adults in a Phase 1 Clinical Trial
Source: J Infect Dis. 2024 Jan 31;230(3):e637–46. doi: 10.1093/infdis/jiae035 (PMC11420805; doi:10.1093/infdis/jiae035)
Supplement: jiae035_Supplementary_Data [file jiae035_supplementary_data.zip › Shaw_JID_SupplementaryMethods_Clean.docx]

# Supplementary Methods

## Study Inclusion criteria

For inclusion in the study, each participant was required to meet the following criteria:

1. Was male or female ≥ 18 to ≤ 49 years of age, who, in the opinion of the Investigator, are in good health based on review of medical history and screening physical examination.
2. In the opinion of the investigator, could and would comply with protocol-mandated follow-up, including all procedures.
3. Adult participant or parent(s)/legal guardian(s) of pediatric participant has provided

written informed consent for participation in this study, including all evaluations and

procedures as specified by this protocol.

1. Had a body mass index (BMI) from ≥ 18 kg/m^2^ to ≤ 35 kg/m^2^.
2. Female participants of non-child-bearing potential could be enrolled in the study.

Non-child-bearing potential was defined as bilateral tubal ligation > 1 year prior to

screening, bilateral oophorectomy, hysterectomy, or menopause. A follicle-stimulating hormone (FSH) level could be measured at the discretion of the Investigator to confirm menopausal status.

1. Female participants of child-bearing potential could be enrolled in the study, if the

participant: 1) had a negative urine pregnancy test at screening and on the day of vaccination, 2) had practiced adequate contraception or abstained from all activities which could lead to pregnancy for 28 days prior to vaccination, 3) had agreed to continue adequate contraception through 3 months following the last injection, and 4) was not currently breastfeeding.

## Study Exclusion criteria

Any of the following was regarded as a criterion for exclusion of a participant from the study:

1. Had Screening laboratory values ≥ Grade 1.
2. Was acutely ill or febrile (temperature ≥ 38.0°C/100.4°F, regardless of route) on the day of the first injection. A participant meeting either of these criteria could be rescheduled for enrolment if the event resolved within the screening window.
3. Had a history of a diagnosis or condition that, in the judgment of the Investigator, could affect study assessment or compromise participant safety, specifically:

- Congenital or acquired immunodeficiency, including human immunodeficiency virus (HIV) infection.
- Chronic hepatitis or suspected active hepatitis.
- Had a bleeding disorder that is considered a contraindication to IM injection or phlebotomy.
- Dermatologic conditions that could affect local solicited AR assessments.
- Any history of allergic or anaphylactic reactions following a vaccination that could require medical intervention.
- Autoimmune disease except for Hashimoto’s disease.

1. Receipt of:

- Inactivated vaccine(s) within 14 days prior to first injection or plans to receive inactivated vaccine(s) within 14 days prior to and through 14 days following each study injection, including but not limited to the following: hepatitis B (hepB), diphtheria, tetanus, and acellular pertussis (DTaP), Haemophilus influenza type B (HiB), pneumococcal conjugate vaccine (PCV13), inactivated poliovirus vaccine (IPV), inactivated influenza vaccine (IIV), hepatitis A (hepA). The exception is any COVID-19 vaccine (regardless of type of vaccine) that became available to participant during the study; efforts should be made to space study vaccinations and COVID-19 vaccinations by at least 7 and preferably 14 days, but COVID-19 vaccinations should not be delayed.
- Live virus vaccine(s) within 28 days prior to first injection or plans to receive live virus vaccine(s) within 28 days prior to and through 28 days following each study injection, including but not limited to the following: measles, mumps, rubella (MMR), varicella (VAR), live-attenuated influenza vaccine (LAIV).
- Systemic immunosuppressants or immune-modifying drugs for > 14 days in total within 6 months prior to the day of enrollment (for corticosteroids, ≥ 2 mg/kg/d or ≥ 20 mg/d prednisone equivalent if the participant weighs > 10 kg). Topical tacrolimus is allowed if not used within 14 days prior to the day of enrollment. Participants could be rescheduled for enrollment if they no longer met this criterion within the Screening window. Inhaled, nasal, and topical steroids were allowed.
- Intravenous blood products (red cells, platelets, Ig) within 3 months prior to enrollment.

1. Had received a drug product containing LNPs within 14 days before enrollment. The

exception is any COVID-19 vaccine (regardless of type of vaccine) that became

available to the participant during the study; efforts should be made to space study

vaccinations and COVID-19 vaccinations by at least 7 and preferably 14 days, but

COVID-19 vaccinations should not be delayed.

1. Had donated ≥ 450 mL of blood products within 28 days of the Screening visit.
2. Had participated in an interventional clinical trial within 28 days prior to the day of enrollment or planned to do so while enrolled in this study.
3. Had a family member or household contact who is an employee of the research center or otherwise involved with the conduct of the study.

## Study Blinding

The study was observer-blind, with blinding of investigators, site personnel, and sponsor such that only designated study personnel responsible for vaccine preparation and administration had access to study treatment assignments. A blinded internal safety monitoring team and an unblinded independent data safety monitoring board provided safety oversight.

## Immunogenicity Assessments

The neutralization assays were conducted as described previously [1]. In brief, a constant amount of virus was mixed with serial dilutions of serum samples as well as the controls and added to a monolayer of HEp-2 cells (ATCC CCL-23). Following an incubation period of 24 hours at 37°C, cells were fixed and immunostained with a monoclonal antibody directed against the RSV F protein followed by a horseradish peroxidase (HRP)-conjugated secondary antibody (Life Technologies) and TrueBlue peroxidase substrate (Seracare). The plates were scanned, and spot counts were determined via ImmunoSpot/BioSpot CTL software. These data were used to determine the dilution of serum that reduced the virus signal by 50%.

A qualified multiplexed assay was used for the detection of IgG antibodies to RSV preF and postF antigens in serum. The assay is based on Luminex technology in which antigen-specific serum antibodies bind directly to the epitopes on antigens covalently conjugated to two distinct Luminex MagPlex-C (Superparamagnetic Carboxylated xMAP) microspheres. Following incubation of the serum samples with antigen coated microspheres, the samples were read on a Bio-Plex 200 (or equivalent) instrument, which identifies the specific Luminex microspheres by their distinct red and infrared fluorescent dye spectral properties. The measured fluorescent signal of the R-Phycoerythrin-conjugated AffiniPure F(ab’)2 Fragment Goat Anti-Human IgG, Fcγ, Fragment Specific secondary antibody is directly proportional to the amount of antigen specific serum IgG antibodies present in the serum samples.

## Sample Size and Analysis Populations

There was no formal sample size calculation performed for this study; however, with 80 younger adult participants receiving the investigational vaccine, there is at least a 98% probability to observe at least 1 participant with an AE at a true 5% AE rate.

Safety was assessed in the Safety Set and Solicited Safety Set. The Safety Set consisted of all randomized participants who received any study injection and was used for analysis of safety data except for the solicited ARs. The Solicited Safety Set consisted of all participants who were randomized and received any study injection and contributed any solicited AR data (ie, had ≥1 postbaseline solicited safety [eDiary] assessment). The Full Analysis Set (FAS) consisted of participants who received any study medication and have baseline data, and ≥1 post injection assessment. The Per-Protocol Set was the primary population for immunogenicity assessments, which consisted of all FAS participants who complied with the injection schedule and the timings of immunogenicity blood sampling and had no major protocol violations that impacted immune response.

**References**

1. Zielinska E, Liu D, Wu HY, Quiroz J, Rappaport R, Yang DP. Development of an improved microneutralization assay for respiratory syncytial virus by automated plaque counting using imaging analysis. Virol J **2005**; 2:84.
