# Supplementary material for: Safety, Tolerability, and Immunogenicity of an mRNA-Based Respiratory Syncytial Virus Vaccine in Healthy Young Adults in a Phase 1 Clinical Trial
Source: J Infect Dis. 2024 Jan 31;230(3):e637–46. doi: 10.1093/infdis/jiae035 (PMC11420805; doi:10.1093/infdis/jiae035)
Supplement: jiae035_Supplementary_Data [file jiae035_supplementary_data.zip › Shaw_Supplementary_Figure1.docx]

## Supplementary Figure 1. Scatter Plot of RSV Neutralizing Titer at Baseline Versus Fold Rise at 1 Month (Per Protocol Set) for (A) RSV-A and (B) RSV-B.
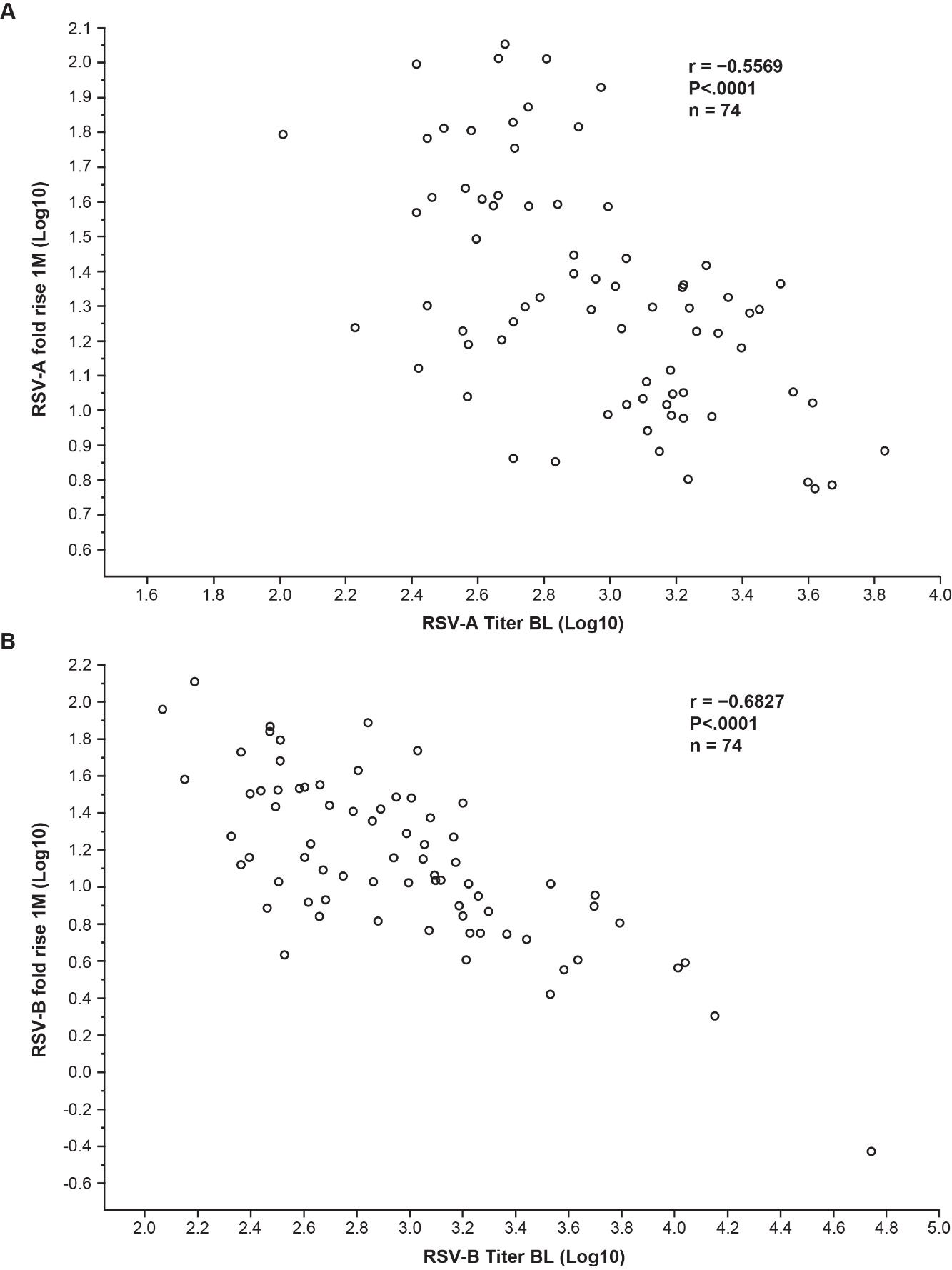


BL, baseline; 1M, 1 month; n, number of XY pairs; r, Spearman correlation coefficient
