# Supplementary material for: Safety, Tolerability, and Immunogenicity of an mRNA-Based Respiratory Syncytial Virus Vaccine in Healthy Young Adults in a Phase 1 Clinical Trial
Source: J Infect Dis. 2024 Jan 31;230(3):e637–46. doi: 10.1093/infdis/jiae035 (PMC11420805; doi:10.1093/infdis/jiae035)
Supplement: jiae035_Supplementary_Data [file jiae035_supplementary_data.zip › Shaw_Supplementary_Figure2.docx]

## Supplementary Figure 2. Scatter Plot of RSV-A Neutralizing Antibody Fold Rise at 1 Month Versus RSV-B Neutralizing Antibody Fold Rise at 1 Month: Per-Protocol Set.*


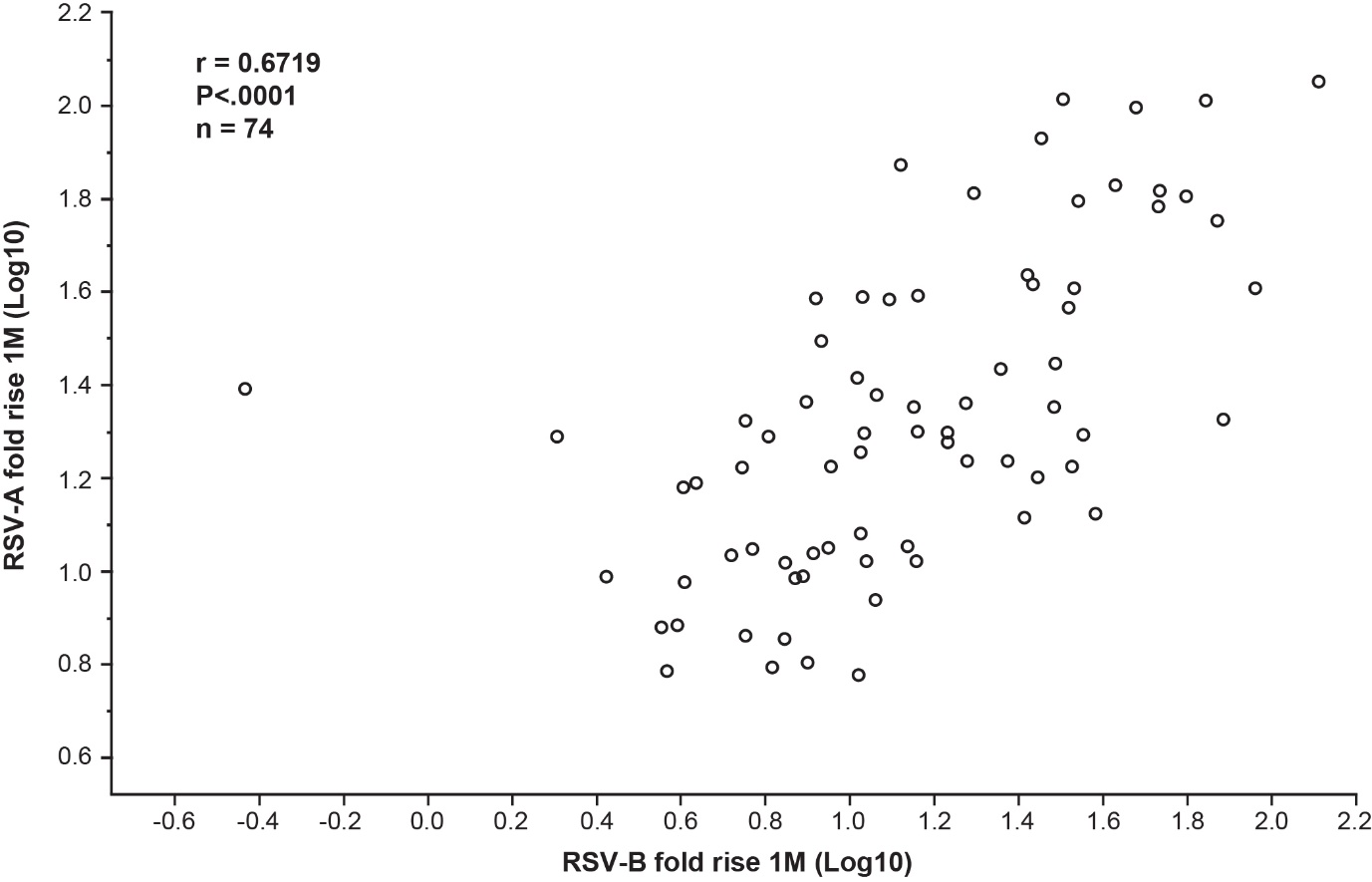
*The Per-Protocol set includes only those individuals dosed with mRNA-1345.

1M, 1 month; n, number of XY pairs; r, Spearman correlation coefficient
