# Supplementary material for: Safety, Tolerability, and Immunogenicity of an mRNA-Based Respiratory Syncytial Virus Vaccine in Healthy Young Adults in a Phase 1 Clinical Trial
Source: J Infect Dis. 2024 Jan 31;230(3):e637–46. doi: 10.1093/infdis/jiae035 (PMC11420805; doi:10.1093/infdis/jiae035)
Supplement: jiae035_Supplementary_Data [file jiae035_supplementary_data.zip › Shaw_Supplementary_Figure3.docx]

##
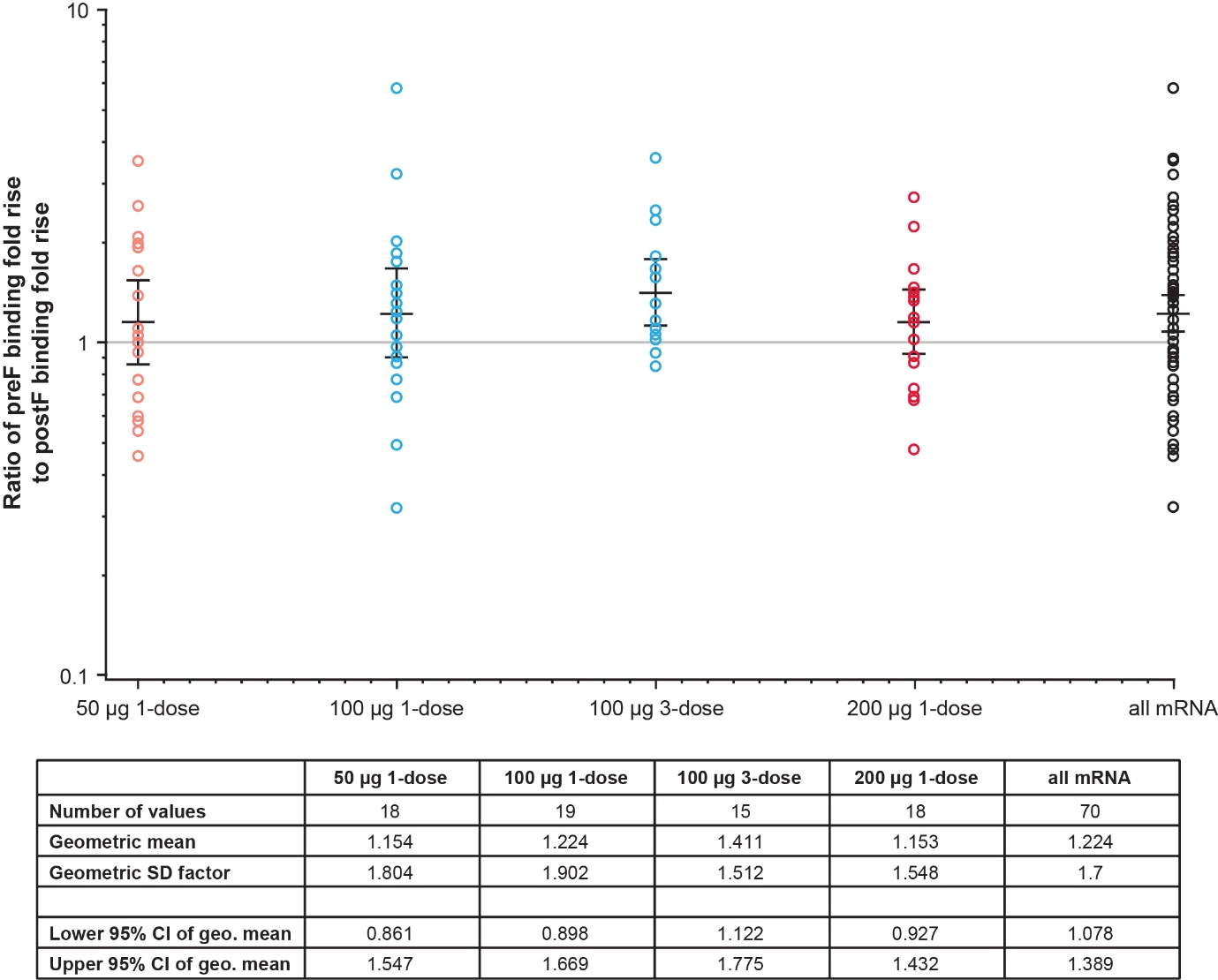
Supplementary Figure 3. Ratio of preF Binding Antibody Fold Rise to postF Binding Antibody Fold Rise at Month 1: Per-Protocol Set.*

*This figure includes only those individuals dosed with mRNA-1345.

CI, confidence interval; postF, postfusion; preF, prefusion; SD, standard deviation
