# Supplementary material for: Safety, Tolerability, and Immunogenicity of an mRNA-Based Respiratory Syncytial Virus Vaccine in Healthy Young Adults in a Phase 1 Clinical Trial
Source: J Infect Dis. 2024 Jan 31;230(3):e637–46. doi: 10.1093/infdis/jiae035 (PMC11420805; doi:10.1093/infdis/jiae035)
Supplement: jiae035_Supplementary_Data [file jiae035_supplementary_data.zip › Shaw_Supplementary_Table1.docx]

## Table S1. Adverse Events of Special Interest

| **Medical Concept** | **Additional Notes** |
| --- | --- |
| Thrombocytopenia | - Platelet counts <150 × 10^9^ - Including but not limited to immune thrombocytopenia, platelet production decreased, thrombocytopenia, thrombocytopenic purpura, thrombotic thrombocytopenic purpura, or hemolysis, elevated liver enzymes, and low platelet count (HELLP) syndrome |
| New onset of or worsening of the following neurologic diseases | - Guillain-Barre Syndrome - Acute disseminated encephalomyelitis (ADEM) - Idiopathic peripheral facial nerve palsy (Bell’s palsy) - Seizures including but not limited to febrile seizures and/or generalized seizures/convulsions |
| Anaphylaxis | - Anaphylaxis as defined per-protocol - Follow the reporting procedures protocol |
| Myocarditis/Pericarditis | - Myocarditis - Pericarditis - Myopericarditis |
