# Supplementary material for: Safety, Tolerability, and Immunogenicity of an mRNA-Based Respiratory Syncytial Virus Vaccine in Healthy Young Adults in a Phase 1 Clinical Trial
Source: J Infect Dis. 2024 Jan 31;230(3):e637–46. doi: 10.1093/infdis/jiae035 (PMC11420805; doi:10.1093/infdis/jiae035)
Supplement: jiae035_Supplementary_Data [file jiae035_supplementary_data.zip › Shaw_Supplementary_Table2.docx]

## Table S2. Solicited Local and Systemic ARs^a^

|  | | **1-Dose Group** | | | | **3-Dose Group** | | | | | | | |
| --- | --- | --- | --- | --- | --- | --- | --- | --- | --- | --- | --- | --- | --- |
|  | | **Placebo** | **mRNA-1345**  **50 μg** | **mRNA-1345**  **100 μg** | **mRNA-1345**  **200 μg** | **Placebo** | | | **mRNA-1345**  **100 μg** | | | |  |
|  | |  |  |  |  | **Dose 1** | **Dose 2** | **Dose 3** | **Dose 1** | | **Dose 2** | **Dose 3** |  |
|  | | **(n=15) ^b^** | **(n=19)^b^** | **(n=20)^b^** | **(n=20)^b^** | **(n=5)^b^** | **(n=4)^b^** | **(n=3)^b^** | **(n=20)^b^** | | **(n=16)^b^** | **(n=15)^b^** |  |
| n (%)^c^ |  |  | |  |  |  |  |  |  |  | |  |  |
| Solicited local AR, N^d^ | | 15 | 19 | 20 | 20 | 5 | 3 | 3 | 20 | | 16 | 14 |  |
| Any solicited local AR | | 0 | 14 (73.7) | 18 (90.0) | 20 (100.0) | 0 | 0 | 0 | 19 (95.0) | | 14 (87.5) | 12 (85.7) |  |
| Grade 1 | | 0 | 9 (47.4) | 14 (70.0) | 7 (35.0) | 0 | 0 | 0 | 13 (65.0) | | 11 (68.8) | 8 (57.1) |  |
| Grade 2 | | 0 | 4 (21.1) | 4 (20.0) | 11 (55.0) | 0 | 0 | 0 | 5 (25.0) | | 3 (18.8) | 3 (21.4) |  |
| Grade 3 | | 0 | 1 (5.3) | 0 | 2 (10.0) | 0 | 0 | 0 | 1 (5.0) | | 0 | 1 (7.1) |  |
| Erythema | | 15 | 19 | 20 | 20 | 5 | 3 | 3 | 20 | | 16 | 14 |  |
| Any | | 0 | 1 (5.3) | 3 (15.0) | 3 (15.0) | 0 | 0 | 0 | 3 (15.0) | | 2 (12.5) | 0 |  |
| Grade 1 | | 0 | 1 (5.3) | 1 (5.0) | 2 (10.0) | 0 | 0 | 0 | 0 | | 0 | 0 |  |
| Grade 2 | | 0 | 0 | 2 (10.0) | 1 (5.0) | 0 | 0 | 0 | 3 (15.0) | | 2 (12.5) | 0 |  |
| Grade 3 | | 0 | 0 | 0 | 0 | 0 | 0 | 0 | 0 | | 0 | 0 |  |
| Pain | | 15 | 19 | 20 | 20 | 5 | 3 | 3 | 20 | | 16 | 14 |  |
| Any | | 0 | 14 (73.7) | 18 (90.0) | 20 (100.0) | 0 | 0 | 0 | 19 (95.0) | | 14 (87.5) | 12 (85.7) |  |
| Grade 1 | | 0 | 10 (52.6) | 16 (80.0) | 7 (35.0) | 0 | 0 | 0 | 15 (75.0) | | 11 (68.8) | 8 (57.1) |  |
| Grade 2 | | 0 | 3 (15.8) | 2 (10.0) | 11 (55.0) | 0 | 0 | 0 | 3 (15.0) | | 3 (18.8) | 3 (21.4) |  |
| Grade 3 | | 0 | 1 (5.3) | 0 | 2 (10.0) | 0 | 0 | 0 | 1 (5.0) | | 0 | 1 (7.1) |  |
| Swelling | | 15 | 19 | 20 | 20 | 5 | 3 | 3 | 20 | | 16 | 14 |  |
| Any | | 0 | 2 (10.5) | 3 (15.0) | 1 (5.0) | 0 | 0 | 0 | 4 (20.0) | | 1 (6.3) | 1 (7.1) |  |
| Grade 1 | | 0 | 1 (5.3) | 2 (10.0) | 0 | 0 | 0 | 0 | 2 (10.0) | | 0 | 1 (7.1) |  |
| Grade 2 | | 0 | 1 (5.3) | 1 (5.0) | 1 (5.0) | 0 | 0 | 0 | 2 (10.0) | | 1 (6.3) | 0 |  |
| Grade 3 | | 0 | 0 | 0 | 0 | 0 | 0 | 0 | 0 | | 0 | 0 |  |
| Solicited systemic AR, N^d^ | | 15 | 19 | 20 | 20 | 5 | 3 | 3 | 20 | | 16 | 14 |  |
| Any solicited systemic AR | | 6 (40.0) | 11 (57.9) | 14 (70.0) | 20 (100.0) | 2 (40.0) | 0 | 1 (33.3) | 18 (90.0) | | 13 (81.3) | 9 (64.3) |  |
| Grade 1 | | 5 (33.3) | 8 (42.1) | 7 (35.0) | 3 (15.0) | 2 (40.0) | 0 | 1 (33.3) | 7 (35.0) | | 5 (31.3) | 3 (21.4) |  |
| Grade 2 | | 1 (6.7) | 2 (10.5) | 6 (30.0) | 11 (55.0) | 0 | 0 | 0 | 10 (50.0) | | 5 (31.3) | 5 (35.7) |  |
| Grade 3 | | 0 | 1 (5.3) | 1 (5.0) | 6 (30.0) | 0 | 0 | 0 | 1 (5.0) | | 3 (18.8) | 1 (7.1) |  |
| Arthralgia | | 15 | 19 | 20 | 20 | 5 | 3 | 3 | 20 | | 16 | 14 |  |
| Any | | 0 | 2 (10.5) | 6 (30.0) | 15 (75.0) | 1 (20.0) | 0 | 0 | 7 (35.0) | | 9 (56.3) | 5 (35.7) |  |
| Grade 1 | | 0 | 2 (10.5) | 3 (15.0) | 6 (30.0) | 1 (20.0) | 0 | 0 | 6 (30.0) | | 7 (43.8) | 3 (21.4) |  |
| Grade 2 | | 0 | 0 | 3 (15.0) | 7 (35.0) | 0 | 0 | 0 | 1 (5.0) | | 1 (6.3) | 1 (7.1) |  |
| Grade 3 | | 0 | 0 | 0 | 2 (10.0) | 0 | 0 | 0 | 0 | | 1 (6.3) | 1 (7.1) |  |
| Chills | | 15 | 19 | 20 | 20 | 5 | 3 | 3 | 20 | | 16 | 14 |  |
| Any | | 0 | 5 (26.3) | 5 (25.0) | 19 (95.0) | 0 | 0 | 0 | 10 (50.0) | | 7 (43.8) | 6 (42.9) |  |
| Grade 1 | | 0 | 3 (15.8) | 2 (10.0) | 5 (25.0) | 0 | 0 | 0 | 4 (20.0) | | 2 (12.5) | 3 (21.4) |  |
| Grade 2 | | 0 | 2 (10.5) | 3 (15.0) | 14 (70.0) | 0 | 0 | 0 | 6 (30.0) | | 5 (31.3) | 3 (21.4) |  |
| Grade 3 | | 0 | 0 | 0 | 0 | 0 | 0 | 0 | 0 | | 0 | 0 |  |
| Fatigue | | 15 | 19 | 20 | 20 | 5 | 3 | 3 | 20 | | 16 | 14 |  |
| Any | | 3 (20.0) | 6 (31.6) | 10 (50.0) | 18 (90.0) | 1 (20.0) | 0 | 0 | 12 (60.0) | | 10 (62.5) | 5 (35.7) |  |
| Grade 1 | | 3 (20.0) | 4 (21.1) | 8 (40.0) | 4 (20.0) | 1 (20.0) | 0 | 0 | 7 (35.0) | | 6 (37.5) | 3 (21.4) |  |
| Grade 2 | | 0 | 2 (10.5) | 1 (5.0) | 9 (45.0) | 0 | 0 | 0 | 5 (25.0) | | 4 (25.0) | 2 (14.3) |  |
| Grade 3 | | 0 | 0 | 1 (5.0) | 5 (25.0) | 0 | 0 | 0 | 0 | | 0 | 2 (7.1) |  |
| Fever | | 15 | 19 | 20 | 20 | 5 | 3 | 3 | 20 | | 16 | 14 |  |
| Any | | 0 | 2 (10.5) | 0 | 6 (30.0) | 0 | 0 | 0 | 7 (35.0) | | 1 (6.3) | 0 |  |
| Grade 1 | | 0 | 1 (5.3) | 0 | 3 (15.0) | 0 | 0 | 0 | 7 (35.0) | | 0 | 0 |  |
| Grade 2 | | 0 | 1 (5.3) | 0 | 3 (15.0) | 0 | 0 | 0 | 0 | | 1 (6.3) | 0 |  |
| Grade 3 | | 0 | 0 | 0 | 0 | 0 | 0 | 0 | 0 | | 0 | 0 |  |
| Headache | | 15 | 19 | 20 | 20 | 5 | 3 | 3 | 20 | | 16 | 14 |  |
| Any | | 4 (26.7) | 7 (36.8) | 6 (30.0) | 19 (95.0) | 1 (20.0) | 0 | 1 (33.3) | 11 (55.0) | | 11 (68.8) | 7 (50.0) |  |
| Grade 1 | | 3 (20.0) | 6 (31.6) | 4 (20.0) | 7 (35.0) | 1 (20.0) | 0 | 1 (33.3) | 8 (40.0) | | 5 (31.3) | 5 (35.7) |  |
| Grade 2 | | 1 (6.7) | 0 | 2 (10.0) | 10 (50.0) | 0 | 0 | 0 | 3 (15.0) | | 4 (25.0) | 2 (14.3) |  |
| Grade 3 | | 0 | 1 (5.3) | 0 | 2 (10.0) | 0 | 0 | 0 | 0 | | 2 (12.5) | 0 |  |
| Lymphadenopathy | | 15 | 19 | 20 | 20 | 5 | 3 | 3 | 20 | | 16 | 14 |  |
| Any | | 0 | 2 (10.5) | 4 (20.0) | 6 (30.0) | 0 | 0 | 0 | 6 (30.0) | | 5 (31.3) | 0 |  |
| Grade 1 | | 0 | 2 (10.5) | 4 (20.0) | 5 (25.0) | 0 | 0 | 0 | 4 (20.0) | | 4 (25.0) | 0 |  |
| Grade 2 | | 0 | 0 | 0 | 0 | 0 | 0 | 0 | 2 (10.0) | | 1 (6.3) | 0 |  |
| Grade 3 | | 0 | 0 | 0 | 1 (5.0) | 0 | 0 | 0 | 0 | | 0 | 0 |  |
| Myalgia | | 15 | 19 | 20 | 20 | 5 | 3 | 3 | 20 | | 16 | 14 |  |
| Any | | 2 (13.3) | 6 (31.6) | 7 (35.0) | 18 (90.0) | 1 (20.0) | 0 | 0 | 11 (55.0) | | 11 (68.8) | 6 (42.9) |  |
| Grade 1 | | 2 (13.3) | 4 (21.1) | 3 (15.0) | 3 (15.0) | 1 (20.0) | 0 | 0 | 2 (10.0) | | 5 (31.3) | 2 (14.3) |  |
| Grade 2 | | 0 | 2 (10.5) | 3 (15.0) | 11 (55.0) | 0 | 0 | 0 | 8 (40.0) | | 5 (31.3) | 3 (21.4) |  |
| Grade 3 | | 0 | 0 | 1 (5.0) | 4 (20.0) | 0 | 0 | 0 | 1 (5.0) | | 1 (6.3) | 1 (7.1) |  |
| Nausea/Vomiting | | 15 | 19 | 20 | 20 | 5 | 3 | 3 | 20 | | 16 | 14 |  |
| Any | | 2 (13.3) | 3 (15.8) | 5 (25.0) | 11 (55.0) | 0 | 0 | 1 (33.3) | 6 (30.0) | | 7 (43.8) | 4 (28.6) |  |
| Grade 1 | | 2 (13.3) | 3 (15.8) | 3 (15.0) | 10 (50.0) | 0 | 0 | 1 (33.3) | 5 (25.0) | | 6 (37.5) | 3 (21.4) |  |
| Grade 2 | | 0 | 0 | 2 (10.0) | 1 (5.0) | 0 | 0 | 0 | 1 (5.0) | | 1 (6.3) | 1 (7.1) |  |
| Grade 3 | | 0 | 0 | 0 | 0 | 0 | 0 | 0 | 0 | | 0 | 0 |  |

AR, adverse reaction.

^a^Participants are counted only once in each category.

^b^Number of participants in the Solicited Safety Set.

^c^Number (%) of participants in each group reporting the event, unless otherwise specified.

^d^Number of participants in the Solicited Safety Set who submitted any data for the event.
