# Supplementary material for: Safety, Tolerability, and Immunogenicity of an mRNA-Based Respiratory Syncytial Virus Vaccine in Healthy Young Adults in a Phase 1 Clinical Trial
Source: J Infect Dis. 2024 Jan 31;230(3):e637–46. doi: 10.1093/infdis/jiae035 (PMC11420805; doi:10.1093/infdis/jiae035)
Supplement: jiae035_Supplementary_Data [file jiae035_supplementary_data.zip › Shaw_Supplementary_Table3.docx]

## Table S3. All Unsolicited AEs (Safety Set^a^)

|  | **1-Dose Group** | | | | **3-Dose Group** | | | | | | | | |
| --- | --- | --- | --- | --- | --- | --- | --- | --- | --- | --- | --- | --- | --- |
|  | **Placebo** | **mRNA-1345**  **50 μg** | **mRNA-1345 100 μg** | **mRNA-1345 200 μg** | **Placebo** | | | | **mRNA-1345 100 μg** | | | |  |
|  |  |  |  |  | **Dose 1** | | **Dose 2** | **Dose 3** | **Dose 1** | **Dose 2** | | **Dose 3** |  |
|  | **(n=15) ^b^** | **(n=19)^b^** | **(n=20)^b^** | **(n=20)^b^** | **(n=5)^b^** | | **(n=4)^b^** | **(n=3)^b^** | **(n=20)^b^** | **(n=16)^b^** | | **(n=15)^b^** |  |
| n (%)^c^ |  |  |  |  |  |  | |  |  | |  |  |  |
| All unsolicited TEAEs, N | 15 | 2 | 2 | 27 | 0 | | 0 | 0 | 8 | 4 | | 17^d^ |  |
| Participants reporting unsolicited TEAEs | 5 (33.3) | 2 (10.5) | 2 (10.0) | 15 (75.0) | 0 | | 0 | 0 | 6 (30.0) | 1 (6.3) | | 3 (20.0) |  |
| Headache | 4 (26.7) | 0 | 0 | 2 (10.0) | 0 | | 0 | 0 | 2 (10.0) | 0 | | 1 (6.7) |  |
| Arthralgia | 0 | 0 | 0 | 1 (5.0) |  | | 0 | 0 | 1 (5.0) | 0 | | 0 |  |
| Injection site erythema | 0 | 0 | 0 | 0 | 0 | | 0 | 0 | 1 (5.0) | 0 | | 0 |  |
| Injection site  induration | 0 | 0 | 1 (5.0) | 0 | 0 | | 0 | 0 | 1 (5.0) | 0 | | 0 |  |
| Lymphadenopathy | 0 | 0 | 0 | 0 | 0 | | 0 | 0 | 1 (5.0) | 0 | | 0 |  |
| Activated partial  thromboplastin  time prolonged | 0 | 0 | 1 (5.0) | 0 | 0 | | 0 | 0 | 0 | 0 | | 0 |  |
| Aphthous ulcer | 0 | 0 | 0 | 2 (10.0) | 0 | | 0 | 0 | 0 | 0 | | 0 |  |
| Ear pain | 0 | 0 | 0 | 1 (5.0) | 0 | | 0 | 0 | 0 | 0 | | 0 |  |
| Injection site bruising | 0 | 0 | 0 | 1 (5.0) | 0 | | 0 | 0 | 0 | 0 | | 0 |  |
| Injection site pain | 0 | 2 (10.5) | 0 | 1 (5.0) | 0 | | 0 | 0 | 0 | 0 | | 0 |  |
| Injection site pruritus | 0 | 0 | 0 | 1 (5.0) | 0 | | 0 | 0 | 0 | 0 | | 0 |  |
| Musculoskeletal  stiffness | 1 (6.7) | 0 | 0 | 1 (5.0) | 0 | | 0 | 0 | 0 | 0 | | 0 |  |
| Palpitations | 0 | 0 | 0 | 1 (5.0) | 0 | | 0 | 0 | 0 | 1 (6.3) | | 1 (6.7) |  |
| Acute sinusitis | 1 (6.7) | 0 | 0 | 1 (5.0) | 0 | | 0 | 0 | 0 | 0 | | 0 |  |
| Skin laceration | 1 (6.7) | 0 | 0 | 0 | 0 | | 0 | 0 | 1 (5.0) | 0 | | 0 |  |
| Dyspnea | 0 | 0 | 0 | 2 (10.0) | 0 | | 0 | 0 | 0 | 0 | | 0 |  |
| Nasopharyngitis | 0 | 0 | 0 | 2 (10.0) | 0 | | 0 | 0 | 0 | 0 | | 0 |  |
| Allergy to arthropod sting | 0 | 0 | 0 | 1 (5.0) | 0 | | 0 | 0 | 0 | 0 | | 0 |  |
| Anxiety | 0 | 0 | 0 | 1 (5.0) | 0 | | 0 | 0 | 0 | 0 | | 0 |  |
| Diarrhea | 0 | 0 | 0 | 1 (5.0) | 0 | | 0 | 0 | 0 | 0 | | 0 |  |
| Ear pain | 0 | 0 | 0 | 1 (5.0) | 0 | | 0 | 0 | 0 | 0 | | 0 |  |
| Fatigue | 0 | 0 | 0 | 1 (5.0) | 0 | | 0 | 0 | 0 | 0 | | 1 (6.7) |  |
| Migraine | 0 | 0 | 0 | 1 (5.0) | 0 | | 0 | 0 | 0 | 0 | | 0 |  |
| Pharynigitis | 0 | 0 | 0 | 1 (5.0) | 0 | | 0 | 0 | 0 | 0 | | 0 |  |
| Pyrexia | 0 | 0 | 0 | 1 (5.0) | 0 | | 0 | 0 | 0 | 0 | | 0 |  |
| Testicular pain | 0 | 0 | 0 | 1 (5.0) | 0 | | 0 | 0 | 0 | 0 | | 0 |  |
| Urticaria | 0 | 0 | 0 | 1 (5.0) | 0 | | 0 | 0 | 0 | 0 | | 0 |  |
| Wound | 0 | 0 | 0 | 0 | 0 | | 0 | 0 | 1 (5.0) | 0 | | 0 |  |
| Limb injury | 1 (6.7) | 0 | 0 | 0 | 0 | | 0 | 0 | 0 | 0 | | 0 |  |
| Morton’s neuralgia | 1 (6.7) | 0 | 0 | 0 | 0 | | 0 | 0 | 0 | 0 | | 0 |  |
| Sensory disturbance | 1 (6.7) | 0 | 0 | 0 | 0 | | 0 | 0 | 0 | 0 | | 0 |  |
| Sinusitis | 1 (6.7) | 0 | 0 | 0 | 0 | | 0 | 0 | 0 | 0 | | 0 |  |
| Toothache | 1 (6.7) | 0 | 0 | 0 | 0 | | 0 | 0 | 0 | 0 | | 0 |  |
| Upper respiratory  tract infection | 1 (6.7) | 0 | 0 | 0 | 0 | | 0 | 0 | 0 | 0 | | 0 |  |
| Depression | 0 | 0 | 0 | 0 | 0 | | 0 | 0 | 0 | 1 (6.3) | | 0 |  |
| Increased appetite | 0 | 0 | 0 | 0 | 0 | | 0 | 0 | 0 | 1 (6.3) | | 0 |  |
| Myalgia | 0 | 0 | 0 | 0 | 0 | | 0 | 0 | 0 | 1 (6.3) | | 0 |  |
| Nausea | 0 | 0 | 0 | 0 | 0 | | 0 | 0 | 0 | 0 | | 2 (13.3) |  |
| Affective disorder | 0 | 0 | 0 | 0 | 0 | | 0 | 0 | 0 | 0 | | 1 (6.7) |  |
| Back pain | 0 | 0 | 0 | 0 | 0 | | 0 | 0 | 0 | 0 | | 1 (6.7) |  |
| Blood pressure  systolic increased | 0 | 0 | 0 | 0 | 0 | | 0 | 0 | 0 | 0 | | 1 (6.7) |  |
| Chest discomfort | 0 | 0 | 0 | 0 | 0 | | 0 | 0 | 0 | 0 | | 1 (6.7) |  |
| Costochondritis | 0 | 0 | 0 | 0 | 0 | | 0 | 0 | 0 | 0 | | 1 (6.7) |  |
| Dizziness | 0 | 0 | 0 | 0 | 0 | | 0 | 0 | 0 | 0 | | 1 (6.7) |  |
| Supraventricular extrasystoles | 0 | 0 | 0 | 0 | 0 | | 0 | 0 | 0 | 0 | | 1 (6.7) |  |
| Tachycardia | 0 | 0 | 0 | 0 | 0 | | 0 | 0 | 0 | 0 | | 1 (6.7) |  |
| Tremor | 0 | 0 | 0 | 0 | 0 | | 0 | 0 | 0 | 0 | | 1 (6.7) |  |
| White blood cell  count increased | 0 | 0 | 0 | 0 | 0 | | 0 | 0 | 0 | 0 | | 1 (6.7) |  |

TEAE, treatment-emergent adverse event.

^a^Participants are counted only once in each category.

^b^Number of participants in the Safety Set who had study vaccination.

^c^Number (%) of participants in each group reporting the event, unless otherwise specified.

^d^Two subjects in the 3-dose cohort experienced the same adverse event twice after dose 3.
