# Supplementary material for: Safety, Tolerability, and Immunogenicity of an mRNA-Based Respiratory Syncytial Virus Vaccine in Healthy Young Adults in a Phase 1 Clinical Trial
Source: J Infect Dis. 2024 Jan 31;230(3):e637–46. doi: 10.1093/infdis/jiae035 (PMC11420805; doi:10.1093/infdis/jiae035)
Supplement: jiae035_Supplementary_Data [file jiae035_supplementary_data.zip › Shaw_Supplementary_Table4.docx]

## Table S4. Neutralizing Antibody Titers

|  | **1-Dose Group** | | | | **3 -Dose Group** | |
| --- | --- | --- | --- | --- | --- | --- |
|  | **Placebo** | **mRNA-1345 50 μg** | **mRNA-1345 100 μg** | **mRNA-1345 200 μg** | **Placebo** | **mRNA-1345 100 μg** |
|  | **(n=15)** | **(n=18)** | **(n=19)** | **(n=19)** | **(n=4)** | **(n=19)** |
| **RSV-A Neutralization (IU/mL)** | |  |  |  |  |  |
| Baseline, n | 15 | 18 | 19 | 19 | 4 | 19 |
| GMT (95% CI) | 1581 (1113.2, 2245.4) | 981.7 (688.4, 1399.8) | 750.1 (472.8, 1190) | 1252.6 (767.5, 2044.3) | 2054.7 (1262.7, 3343.4) | 811.9 (525.6, 1254.1) |
| Month 1, n | 15 | 18 | 19 | 18 | 4 | 19 |
| GMT (95% CI) | 1461 (941.7, 2266.6) | 20140.7 (13749.6, 29502.4) | 16687.8 (11984.9, 23236.2) | 22312.3 (15470.8, 32179.3) | 1988.9 (930.6, 4251.1) | 19111.9 (29495.1, 12383.9) |
| GMFR (95% CI) | 0.9 (0.8, 1.1) | 20.5 (13.6, 30.9) | 22.3 (14.87, 33.5) | 20.0 (14.0, 28.6) | 1.0 (0.7, 1.4) | 23.5 (16.1, 34.3) |
| Month 2, n | 14 | 18 | 19 | 17 | 4 | 15 |
| GMT (95% CI) | 1321.8 (928.2, 1882.3) | 17184.7 (11128.9, 26535.8) | 12624.2 (8407.6, 18955.4) | 17390.2 (10457.9, 28917.8) | 1564.6 (665.8, 3676.6) | 11856 (5981.3, 23500.7) |
| GMFR (95% CI) | 0.9 (0.7, 1.1) | 17.5 (11.0, 27.9) | 16.8 (10.1, 28.1) | 13.9 (9.8, 19.8) | 0.8 (0.3, 2.0) | 15.3 (9.2, 25.4) |
| Month 3, n | 13 | 18 | 19 | 17 | 3 | 13 |
| GMT (95% CI) | 1426.1 (926.5, 2195.2) | 15484.6 (10242.6, 23409.4) | 8838.7 (6448.5, 12114.9) | 12581.7 (8142.4, 19441.4) | 1400.5 (555.1, 3533.8) | 17802.2 (9821.8, 32267) |
| GMFR (95% CI) | 1.0 (0.8, 1.2) | 15.8 (10.7, 23.3) | 11.8 (7.8, 17.9) | 9.3 (6.0, 14.6) | 0.7 (0.4, 1.5) | 21.2 (11.5, 39.0) |
| Month 4, n | 13 | 18 | 17 | 16 | 3 | 12 |
| GMT (95% CI) | 1230.2 (815.4, 1856) | 13991.8 (9014.2, 21718.1) | 7034.8 (5109.9, 9684.7) | 14692.4 (9907.9, 21787.4) | 1940.4 (297.7, 12646.6) | 12571.6 (6782.8, 23300.7) |
| GMFR (95% CI) | 0.9 (0.7, 1.1) | 14.3 (9.1, 22.3) | 8.7 (5.3, 14.3) | 9.6 (6.7, 13.8) | 1.0 (0.2, 5.3) | 17.2 (10.6, 28.0) |
| Month 5, n | 14 | 18 | 19 | 16 | 3 | 13 |
| GMT (95% CI) | 1378.9 (857.8, 2216.6) | 9561.8 (5982.5, 15282.5) | 5582.9 (4028.9, 7736.2) | 14375.8 (9374.3, 22045.8) | 1909.1 (522.8, 6970.7) | 10500.2 (6144.7, 17942.8) |
| GMFR (95% CI) | 1.0 (0.7, 1.3) | 9.7 (6.1, 15.6) | 7.4 (4.9, 11.2) | 9.2 (6.8, 12.5) | 1.0 (0.1, 7.5) | 15.6 (9.5, 25.4) |
| Month 6, n | 13 | 18 | 19 | 17 | 3 | 13 |
| GMT (95% CI) | 1334.8 (902.7, 1973.7) | 9448 (6169.7, 14468.4) | 5232.8 (3684.1, 7432.5) | 11516.4 (6681.9, 19848.7) | 1345.1 (363.4, 4978.4) | 13287.2 (7992.9, 22088.2) |
| GMFR (95% CI) | 1.0 (0.8, 1.2) | 9.6 (6.3, 14.8) | 7.0 (4.6, 10.5) | 8.4 (5.9, 12.0) | 0.7 (0.2, 3.4) | 19.7 (12.8, 30.3) |
| Month 10, n | NA | NA | NA | NA | 3 | 12 |
| GMT (95% CI) | NA | NA | NA | NA | 1103.5 (776.9, 1567.4) | 8669.8 (5301.3, 14178.7) |
| GMFR (95% CI) | NA | NA | NA | NA | 0.6 (0.4, 1.0) | 12.6 (8.0, 19.8) |
| **RSV-B Neutralization (IU/mL)** | |  |  |  |  |  |
| Baseline | 15 | 18 | 19 | 19 | 4 | 19 |
| GMT (95% CI) | 1144.2 (694.9, 1884.1) | 795.3 (532.8, 1187.3) | 1091.6 (560.2, 2127.3) | 1097.4 (533.8, 2255.8) | 1373.7 (448.2, 4210.6) | 842.7 (494.3, 1436.5) |
| Month 1 | 15 | 18 | 19 | 18 | 4 | 19 |
| GMT (95% CI) | 1009.5 (612.3, 1664.5) | 11487.5 (7894.8, 16715.3) | 12741.6 (9388.1, 17293) | 12901.6 (8474.9, 19640.5) | 1640.5 (294.4, 9143.0) | 13496.5 (8379.8, 21737.4) |
| GMFR (95% CI) | 0.9 (0.7, 1.1) | 14.4 (9.6, 21.8) | 11.7 (6.7, 20.3) | 13.9 (8.1, 23.9) | 1.2 (0.6, 2.4) | 16.0 (9.9, 25.9) |
| Month 2, n | 14 | 18 | 19 | 17 | 4 | 15 |
| GMT (95% CI) | 1053.3 (527.6, 2102.6) | 12156.5 (7773,0 19012.1) | 14221.7 (9366.5, 21593.6) | 9374.5 (5178.1, 16971.7) | 2168.5 (411.5, 11428.7) | 15341.5 (10067.7, 23378) |
| GMFR (95% CI) | 0.9 (0.7, 1.3) | 15.3 (9.6, 24.3) | 13.0 (8.1, 20.9) | 8.7 (5.2, 14.6) | 1.6 (0.5, 4.6) | 19.3 (12.3, 30.2) |
| Month 3, n | 13 | 18 | 19 | 17 | 3 | 13 |
| GMT (95% CI) | 939.1 (513.2, 1718.6) | 8380 (5212.3, 13472.9) | 8996.4 (5678.6, 14252.8) | 9007.3 (4360.2, 18607) | 1324.8 (98.6, 17800.2) | 13400.3 (8814.8, 20371.2) |
| GMFR (95% CI) | 0.9 (0.6, 1.4) | 10.5 (6.8, 16.4) | 8.2 (5.3, 13.0) | 7.3 (4.4, 12.4) | 1.2 (0.4, 3.9) | 14.9 (8.7, 25.5) |
| Month 4, n | 13 | 18 | 17 | 16 | 3 | 12 |
| GMT (95% CI) | 874.2 (447.1, 1709.4) | 8255 (5581.9, 12208.0) | 10657 (6790.7, 16724.4) | 8744.3 (4545.8, 16820.5) | 1459.2 (162.8, 13081.1) | 13515.1 (8581.1, 21286) |
| GMFR (95% CI) | 0.8 (0.6, 1.1) | 10.4 (6.5, 16.6) | 8.5 (5.4, 13.5) | 6.4 (3.7, 11.0) | 1.3 (0.7, 2.5) | 16.5 (8.5, 32.2) |
| Month 5, n | 14 | 18 | 19 | 16 | 3 | 13 |
| GMT (95% CI) | 963.4 (571.5, 1624.0) | 8478.6 (5461.9, 13161.5) | 8019.8 (5289.7, 12159.0) | 7313.6 (3859.4, 13859.6) | 1478.9 (79.2, 27622.2) | 14408.6 (8599.6, 24141.7) |
| GMFR (95% CI) | 0.9 (0.7, 1.1) | 10.7 (6.5, 17.6) | 7.4 (4.5, 12.0) | 5.2 (3.3, 8.2) | 1.4 (0.4, 5.2) | 20.2 (12.2, 33.4) |
| Month 6, n | 13 | 18 | 19 | 17 | 3 | 13 |
| GMT (95% CI) | 730.6 (394.3, 1353.8) | 7078.4 (4563.6, 10978.8) | 5769.0 (3878.2, 8581.6) | 6276.6 (3418.9, 11522.9) | 1119.5 (66.8, 18749.5) | 11801.7 (7020.9, 19837.7) |
| GMFR (95% CI) | 0.7 (0.5, 0.9) | 8.9 (5.7, 13.9) | 5.3 (3.5, 7.9) | 5.0 (3.2, 8.0) | 1.0 (0.3, 4.0) | 16.5 (9.4, 29.1) |
| Month 10, n | NA | NA | NA | NA | 3 | 12 |
| GMT (95% CI) | NA | NA | NA | NA | 819.2 (22.2, 30249.3) | 7081.8 (4123.2, 12163.2) |
| GMFR (95% CI) | NA | NA | NA | NA | 0.8 (0.1, 5.9) | 10.7 (5.8, 19.5) |
|  |  |  |  |  |  |  |

GMFR, geometric mean fold rise; GMT, geometric mean titer.
