# Supplementary material for: Safety, Tolerability, and Immunogenicity of an mRNA-Based Respiratory Syncytial Virus Vaccine in Healthy Young Adults in a Phase 1 Clinical Trial
Source: J Infect Dis. 2024 Jan 31;230(3):e637–46. doi: 10.1093/infdis/jiae035 (PMC11420805; doi:10.1093/infdis/jiae035)
Supplement: jiae035_Supplementary_Data [file jiae035_supplementary_data.zip › Shaw_Supplementary_Table5.docx]

## Table S5. Binding Antibody Concentrations

|  | **1-Dose Group** | | | | **3 -Dose Group** | |
| --- | --- | --- | --- | --- | --- | --- |
|  | **Placebo** | **mRNA-1345 50 μg** | **mRNA-1345 100 μg** | **mRNA-1345 200 μg** | **Placebo** | **mRNA-1345 100 μg** |
|  | **(n=15)** | **(n=18)** | **(n=19)** | **(n=19)** | **(n=4)** | **(n=19)** |
| **PreF-Binding Antibody (AU/mL)** | | | | |  |  |
| Baseline, n | 14 | 18 | 19 | 19 | 4 | 15 |
| GMC (95% CI) | 7272.6 (5784.0, 9144.5) | 7467.2 (5552.0, 10043.2) | 5812.9 (4162.1, 8118.4) | 7722.4 (5034.0, 11846.5) | 9290.5 (6403.5, 13479.2) | 6215.6 (4738.9, 8152.4) |
| Month 1, n | 14 | 18 | 19 | 18 | 4 | 15 |
| GMC (95% CI) | 7226.1 (5489.8, 9511.7) | 120491.6 (92412.4, 157102.6) | 125952.6 (103884.6, 152708.3) | 116742.7 (89152.0, 152872.1) | 10307.3 (7281.3, 14590.9) | 135260.0 (95356.2, 191862.2) |
| GMFR (95% CI) | 1.0 (0.9, 1.1) | 16.1 (11.4, 22.8) | 21.7 (15.4, 30.5) | 16.9 (12.4, 22.9) | 1.1 (0.9, 1.4) | 21.8 (16.7, 28.3) |
| Month 2, n | 14 | 18 | 19 | 17 | 4 | 14 |
| GMC (95% CI) | 7659.7 (5931.1, 9892.2) | 98849.5 (75545.8, 129341.8) | 89896.9 (70717.8, 114277.4) | 90612.8 (65451.1, 125447.6) | 9097.5 (7091.1, 11671.6) | 98889.3 (65188.1, 150013.4) |
| GMFR (95% CI) | 1.1 (1.0, 1.1) | 13.2 (9.7, 18.1) | 15.5 (10.8, 22.2) | 11.9 (8.5, 16.7) | 1.0 (0.8, 1.2) | 16.0 (11.6, 22.1) |
| Month 3, n | 13 | 18 | 19 | 17 | 3 | 12 |
| GMC (95% CI) | 7154.4 (5232.9, 9781.4) | 69574.6 (50071.5, 96674.2) | 67912.3 (51691.0, 89224.0) | 70057.9 (49495.6, 99162.5) | 10664.3 (8260.2, 13768.0) | 112481.0 (76987.5, 164337.9) |
| GMFR (95% CI) | 1.0 (0.9, 1.1) | 9.3 (6.4, 13.6) | 11.7 (7.9, 17.2) | 9.3 (6.9, 12.5) | 1.0 (0.8, 1.3) | 17.1 (13.1, 22.4) |
| Month 4, n | 13 | 18 | 17 | 16 | 3 | 12 |
| GMC (95% CI) | 6877.3 (5199.8, 9096.0) | 61182.6 (43795.1, 85473.2) | 58058.4 (43549.8, 77400.6) | 61171.5 (44176.0, 84705.6) | 10317.7 (9299.1, 11447.8) | 103093.5 (66326.3, 160241.9) |
| GMFR (95% CI) | 1.0 (0.9, 1.1) | 8.2 (5.9, 11.3) | 9.7 (6.5, 14.4) | 7.5 (5.3, 10.6) | 1.0 (0.9, 1.2) | 15.3 (10.8, 21.6) |
| Month 5, n | 14 | 18 | 19 | 16 | 3 | 13 |
| GMC (95% CI) | 7077.7 (5307.6, 9438.1) | 57291 (40876.2, 80297.6) | 48121.1 (37794.8, 61268.8) | 59941.7 (46326.6, 77558.0) | 10026.5 (6884.4, 14602.7) | 99738.7 (70499.4, 141104.8) |
| GMFR (95% CI) | 1.0 (0.9, 1.1) | 7.7 (5.5, 10.8) | 8.3 (6.0, 11.4) | 6.5 (4.7, 9.0) | 1.0 (0.6, 1.5) | 16.1 (12.1, 21.3) |
| Month 6, n | 13 | 18 | 19 | 17 | 3 | 13 |
| GMC (95% CI) | 6953.1 (5161.5, 9366.6) | 48392.9 (34510.9, 67858.8) | 40948.1 (31357.1, 53472.6) | 49499.8 (35508.9, 69003.3) | 9958.8 (9347.1, 10610.6) | 83648.6 (56870.6, 123035.3) |
| GMFR (95% CI) | 1.0 (0.9, 1.1) | 6.5 (4.7, 8.9) | 7.0 (5.1, 9.7) | 5.9 (4.4, 7.8) | 1.0 (0.9, 1.0) | 13.5 (10.1, 18.0) |
| Month 10, n | NA | NA | NA | NA | 3 | 12 |
| GMC (95% CI) |  |  |  |  | 8330.4 (3649.2, 19016.7) | 54418.7 (36137.6, 81947.8) |
| GMFR (95% CI) | NA | NA | NA | NA | 0.8 (0.3, 1.9) | 8.4 (5.9, 12.1) |
| **PostF-Binding Antibody (AU/mL)** | | |  |  |  |  |
| Baseline, n | 14 | 18 | 19 | 19 | 4 | 15 |
| GMC (95% CI) | 7788.2 (5463.6, 11101.9) | 7781.5 (5367, 11282.3) | 6372.2 (4670.2, 8694.6) | 9605.3 (5951.6, 15502) | 7751.9 (996.6, 60412.1) | 8569.1 (5972, 12295.8) |
| Month 1, n | 14 | 18 | 19 | 18 | 4 | 15 |
| GMC (95% CI) | 8011.2 (5514.7, 11637.9) | 108811.5 (72461.7, 163395.8) | 112799.6 (82736.5, 153786.4) | 130974.4 (97851.3, 175309.8) | 8495.6 (1077.7, 66969.9) | 132127.9 (84337.9, 206998.2) |
| GMFR (95% CI) | 1.0 (0.9, 1.1) | 14.0 (8.7, 22.5) | 17.7 (11.8, 26.7) | 14.6 (10.8, 19.8) | 1.1 (1.0, 1.3) | 15.4 (11.0, 21.6) |
| Month 2, n | 14 | 18 | 19 | 17 | 4 | 14 |
| GMC (95% CI) | 7984.5 (5469.2, 11656.5) | 85196 (58250, 124606.9) | 75628.1 (51928.5, 110143.8) | 81923.9 (56585.6, 118608.3) | 7997.4 (909.5, 70318.9) | 94125.8 (155811, 56861) |
| GMFR (95% CI) | 1.0 (1.0, 1.1) | 11.0 (7.1, 17.0) | 11.9 (7.7, 18.3) | 9.1 (6.65, 12.41) | 1.0 (0.9, 1.2) | 10.9 (7.4, 15.9) |
| Month 3, n | 13 | 18 | 19 | 17 | 3 | 12 |
| GMC (95% CI) | 7318.3 (5049.8, 10605.9) | 60967 (39795.8, 93401) | 59128 (40040.4, 87314.9) | 61176.8 (42975.5, 87086.9) | 7683.9 (166.3, 354981.2) | 89605.4 (157825.7, 50873.4) |
| GMFR (95% CI) | 1.0 (1.0, 1.1) | 7.8 (4.8, 12.8) | 9.3 (5.9, 14.5) | 6.9 (5.1, 9.4) | 1.1 (0.9, 1.3) | 9.9 (6.5, 15.0) |
| Month 4, n | 13 | 18 | 17 | 16 | 3 | 12 |
| GMC (95% CI) | 8374.5 (5784.3, 12124.5) | 53258.8 (35653.9, 79556.5) | 51435 (33669.1, 78575.2) | 53606.8 (36326.1, 79108) | 7441.6 (150.4, 368224) | 85589.8 (150634, 48631.9) |
| GMFR (95% CI) | 1.1 (1.0, 1.1) | 6.8 (4.5, 10.5) | 7.6 (4.7, 12.1) | 5.6 (4.0, 7.8) | 1.1 (1.0, 1.2) | 9.0 (5.8, 13.9) |
| Month 5, n | 14 | 18 | 19 | 16 | 3 | 13 |
| GMC (95% CI) | 8019.4 (5567.2, 11551.7) | 48311.1 (32332.8, 72185.7) | 41981.1 (27984.5, 62977.9) | 52803.7 (35845.3, 77785.3) | 7352.4 (177.1, 305240.2) | 71562.6 (118406.8, 43250.9) |
| GMFR (95% CI) | 1.0 (1.0, 1.1) | 6.2 (4.1, 9.5) | 6.6 (4.2, 10.3) | 4.5 (3.4, 6.1) | 1.1 (0.9, 1.3) | 8.0 (5.5, 11.6) |
| Month 6, n | 13 | 18 | 19 | 17 | 3 | 13 |
| GMC (95% CI) | 8438.3 (5792.5, 12292.6) | 41471.6 (27763.8, 61947.1) | 34074.2 (23529.9, 49343.5) | 44688.8 (66121.4, 30203.3) | 7024.3 (110.4, 446737.5) | 63113.6 (37586.8, 105976.6) |
| GMFR (95% CI) | 1.1 (1.0, 1.2) | 5.3 (3.5, 8.0) | 5.4 (3.6, 8.0) | 4.2 (3.1, 5.6) | 1.0 (0.7, 1.5) | 7.0 (4.9, 10.2) |
| Month 10, n | NA | NA | NA | NA | 3 | 12 |
| GMC (95% CI) | NA | NA | NA | NA | 5859.6 (134.3, 255601.8) | 43784.2 (24668.6, 77712.5) |
| GMFR (95% CI) | NA | NA | NA | NA | 0.9 (0.4, 1.8) | 4.8 (3.3, 7.0) |

GMFR, geometric mean fold rise; GMC, geometric mean concentration.
